# Psychiatric comorbidities in epilepsy: population co-occurrence, genetic correlations and causal effects

**DOI:** 10.1136/gpsych-2023-101201

**Published:** 2024-01-30

**Authors:** Viktor H Ahlqvist, Christina Dardani, Paul Madley-Dowd, Harriet Forbes, Jessica Rast, Caichen Zhong, Renee M Gardner, Christina Dalman, Kristen Lyall, Craig Newschaffer, Torbjörn Tomson, Michael Lundberg, Daniel Berglind, Neil M Davies, Brian K Lee, Cecilia Magnusson, Dheeraj Rai

**Affiliations:** 1 Department of Global Public Health, Karolinska Institutet, Stockholm, Sweden; 2 Medical Research Council Integrative Epidemiology Unit, Bristol Medical School, University of Bristol, Bristol, UK; 3 Centre for Academic Mental Health, Population Health Sciences, Bristol Medical School, University of Bristol, Bristol, UK; 4 Faculty of Epidemiology and Population Health, London School of Hygiene and Tropical Medicine, London, UK; 5 A.J. Drexel Autism Institute, Drexel University, Philadelphia, PA, USA; 6 Department of Epidemiology and Biostatistics, School of Public Health, Drexel University, Philadelphia, PA, USA; 7 College of Health and Human Development, Pennsylvania State University, State College, PA, USA; 8 Department of Clinical Neuroscience, Karolinska Institutet, Stockholm, Sweden; 9 Centre for Epidemiology and Community Medicine, Region Stockholm, Stockholm, Sweden; 10 K.G. Jebsen Center for Genetic Epidemiology, Department of Public Health and Nursing, Norwegian University of Science and Technology, Trondheim, Norway; 11 Division of Psychiatry, University College London, London, UK; 12 Department of Statistical Sciences, University College London, London, UK; 13 NIHR Biomedical Research Centre, University of Bristol, Bristol, UK; 14 Avon and Wiltshire Partnership, NHS Mental Health Trust, Bristol, UK

**Keywords:** neuropsychiatry, psychiatry

## Abstract

**Background:**

Psychiatric comorbidities are common in patients with epilepsy. Reasons for the co-occurrence of psychiatric conditions and epilepsy remain poorly understood.

**Aim:**

We aimed to triangulate the relationship between epilepsy and psychiatric conditions to determine the extent and possible origins of these conditions.

**Methods:**

Using nationwide Swedish health registries, we quantified the lifetime prevalence of psychiatric disorders in patients with epilepsy. We then used summary data from genome-wide association studies to investigate whether the identified observational associations could be attributed to a shared underlying genetic aetiology using cross-trait linkage disequilibrium score regression. Finally, we assessed the potential bidirectional relationships using two-sample Mendelian randomisation.

**Results:**

In a cohort of 7 628 495 individuals, we found that almost half of the 94 435 individuals diagnosed with epilepsy were also diagnosed with a psychiatric condition in their lifetime (adjusted lifetime prevalence, 44.09%; 95% confidence interval (CI) 43.78% to 44.39%). We found evidence for a genetic correlation between epilepsy and some neurodevelopmental and psychiatric conditions. For example, we observed a genetic correlation between epilepsy and attention-deficit/hyperactivity disorder (r_g_=0.18, 95% CI 0.09 to 0.27, p<0.001)—a correlation that was more pronounced in focal epilepsy (r_g_=0.23, 95% CI 0.09 to 0.36, p<0.001). Findings from Mendelian randomisation using common genetic variants did not support bidirectional effects between epilepsy and neurodevelopmental or psychiatric conditions.

**Conclusions:**

Psychiatric comorbidities are common in patients with epilepsy. Genetic correlations may partially explain some comorbidities; however, there is little evidence of a bidirectional relationship between the genetic liability of epilepsy and psychiatric conditions. These findings highlight the need to understand the role of environmental factors or rare genetic variations in the origins of psychiatric comorbidities in epilepsy.

**WHAT IS ALREADY KNOWN ON THIS TOPIC**

- Although psychiatric comorbidities are common in patients with epilepsy, the reasons for the co-occurrence of psychiatric conditions and epilepsy remain poorly understood.

**WHAT THIS STUDY ADDS**

- Almost half of the 94 435 individuals diagnosed with epilepsy in Sweden were also diagnosed with psychiatric conditions during their lifetime.
- Some psychiatric conditions share an underlying genetic architecture with epilepsy, which may be more pronounced in focal epilepsy.
- Mendelian randomisation analyses did not support the hypothesis that epilepsy causally increases the risk of psychiatric conditions and vice versa.

**HOW THIS STUDY MIGHT AFFECT RESEARCH, PRACTICE OR POLICY**

- The lack of widespread genetic correlation and effects in either direction suggests that environmental factors may explain psychiatric comorbidities in epilepsy.

## Introduction

Epilepsy co-occurs with psychiatric conditions throughout life and contributes to the challenges faced by people with epilepsy. For example, Swedish register-based studies have suggested that the lifetime prevalence of a psychiatric diagnosis among people with epilepsy is as high as 40%, compared with ∼10% of the general population.^1^ Individuals with epilepsy are more likely to have neurodevelopmental disorders (eg, autism, attention-deficit/hyperactivity disorder, tic disorders and obsessive-compulsive disorders) as well as other psychiatric conditions (eg, anorexia nervosa, anxiety, bipolar disorder, depression and schizophrenia). However, the reasons for these co-occurrences remain largely unknown.^2–4^

Few large-scale studies have examined the population-wide comorbidity of epilepsy and psychiatric disorders,^5 6^ possible genetic correlations between comorbidities^7–9^ or the direction of the effects using Mendelian randomisation (MR).^10 11^ Psychiatric comorbidities in epilepsy may be a function of shared genetic aetiology, bidirectional relationships, shared environmental aetiology, chance and artefactual processes or a combination of these.^2^ A better understanding of the mechanisms underlying psychiatric comorbidities in epilepsy may help identify targets for the prevention or treatment of these comorbidities.

It is often suggested that the psychiatric comorbidities in epilepsy are explained by bidirectional relationships,^12^ where psychiatric conditions increase the risk of epilepsy, and vice versa, that epilepsy increases the risk of psychiatric conditions. This notion is supported by a series of neurobiological pathogenic mechanisms (eg, induced abnormalities in cortical and subcortical structures).^13^ Yet, observational studies contributing to this hypothesis^12^ have typically been limited in sample size, liable to confounding factors and/or have been restricted to clinical cohorts with limited generalisability.

In this study, we used novel and standard methods to triangulate the relationship between epilepsy and psychiatric conditions (online supplemental figure 1). First, using Swedish health registers, we characterised the rate of comorbidity of different psychiatric conditions in the largest nationwide cohort of individuals with epilepsy. Second, we investigated whether any comorbidities could be attributed to a shared underlying genetic architecture by assessing genetic correlations using cross-trait linkage disequilibrium score regression.^14 15^ Finally, we examined the possibility of bidirectional effects by using MR, a design that leverages the random assortment of genotypes associated with epilepsy and psychiatric disorders, and allows for the exploration of the direction of effects.^16^

10.1136/gpsych-2023-101201.supp1 Supplementary data

## Methods

All research was performed in accordance with the relevant guidelines and regulations.

### Swedish population-based registries

**Figure 1:**
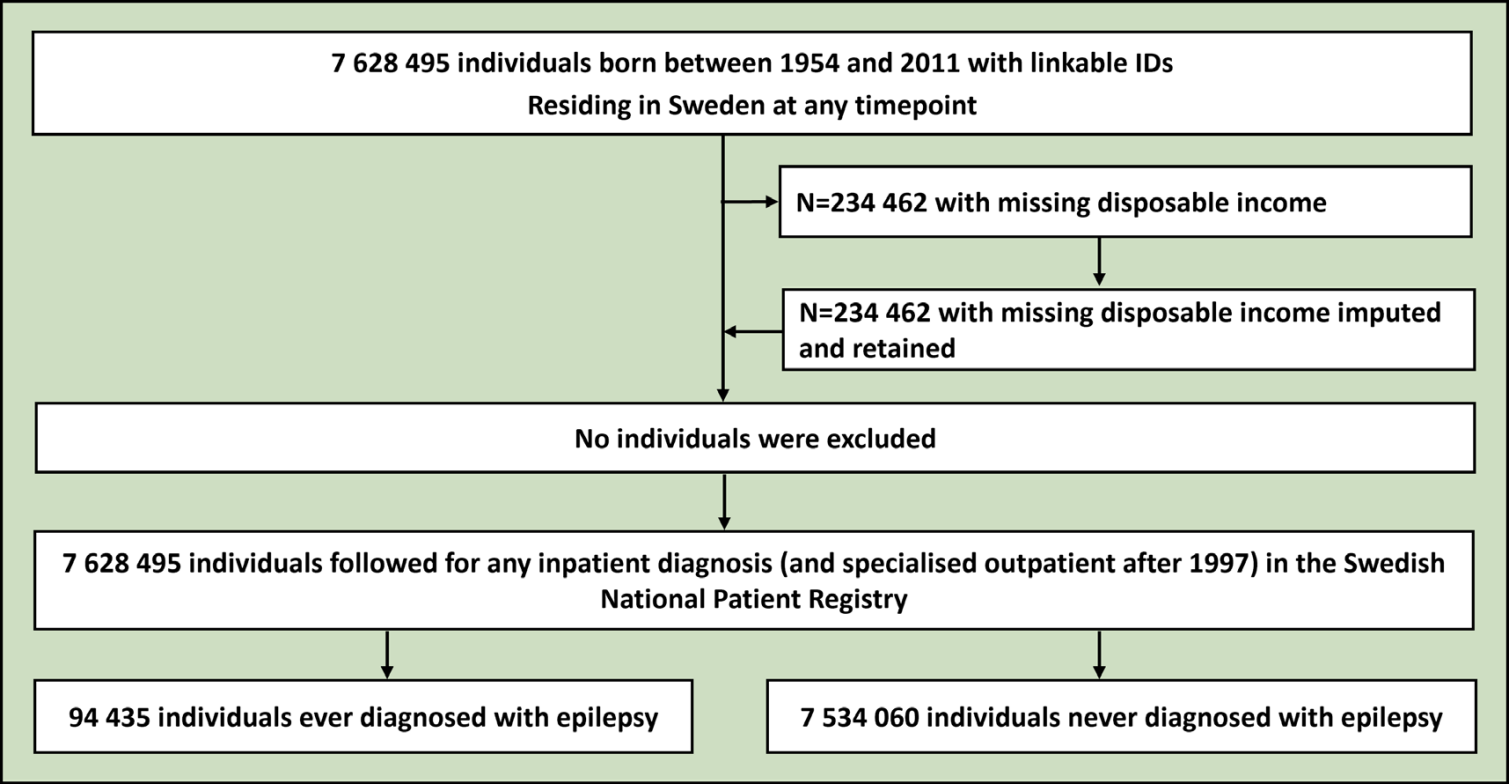
Flowchart depicting the derivation of the analytical sample from the Swedish registry data. No individuals were excluded from the analysis. Those missing disposable income were imputed, as they were either too young to have accumulated any disposable income, or this information was missing. Further details are available in the text.

To estimate the population-level co-occurrence of epilepsy and psychiatric conditions, we derived a nationwide cohort of 7 628 495 individuals born between 1954 and 2011 from the Swedish population registries (figure 1). Using the Total Population Registry and the National Patient Registry (initiation 1969, end of follow-up 2016), we collected information on vital statistics, immigration status (born in or outside Sweden), birth year and any inpatient diagnosis (and specialised outpatient after 1997) of the investigated psychiatric conditions and epilepsy (see online supplemental table 1 for International Classification of Diseases (ICD) 8/9/10 codes). We also identified disposable income quintiles for each year between 1968 and 2016 and retained the highest quintile obtained over a person’s lifetime from the Income and Taxation Registry, which we conceptualised as both a socioeconomic proxy and a measure of resilience to morbidity manifestation. Individuals born after 1996 with missing disposable income values were designated as a distinct category, as they were probably too young to have accumulated disposable income.

The National Patient Registry records the date of diagnosis and not the date of onset. Therefore, we focused solely on estimating lifetime comorbidities of epilepsy and psychiatric disorders.

#### Statistical methods

Logistic regression was used to estimate the crude and conditional odds ratio (OR) and 95% confidence interval (CI) of a lifetime diagnosis of psychiatric conditions among individuals ever diagnosed with epilepsy (n=94 435) compared with those who were never diagnosed (n=7 534 060), controlling for birth year, sex, highest disposable income achieved and immigration status. We also estimated the crude and counterfactual probabilities of the outcomes (holding all covariates at their initial values). Hereafter, we refer to this quantity as the adjusted lifetime prevalence. This is because it captures the estimated prevalence among those with epilepsy if they had the same covariate distribution as those without epilepsy, and vice versa. In the primary analysis, we did not separate the types of epilepsy, as they are often not well characterised in the National Patient Registry and because many people with epilepsy often have multiple seizure types. However, in a complementary analysis, we classified individuals based on the most recent instance in which they received either a focal (n=24 430, 25.9%) or generalised epilepsy diagnosis (n=19 199, 20.3%), although some individuals remained uncharacterised (either unspecific (n=32 468, 34.4%) or were never coded with sufficient detail (n=18 079, 19.1%)). We chose not to classify 259 (0.3%) individuals who were discharged with both focal and generalised ICD codes on their most recent visit. In principle, there should be no missing data in the analysed variables (reporting mandated by Swedish law). However, there was some missing data in disposable income (≈3%), and we treated these as a distinct income category.

### Genome-wide association data sources and phenotypes

Summary statistics were retrieved from the largest European ancestry genome-wide association study (GWAS) of epilepsy performed by the International League Against Epilepsy (ILAE) Consortium on Complex Epilepsies.^7^ In brief, all epilepsy cases were classified according to ILAE standard and all phenotypes were assessed by epilepsy specialists at individual sites. Controls (persons without epilepsy) were derived from the available population-based datasets within the Consortium on Complex Epilepsies and partially screened for the presence of neurological conditions. Extended phenotypic information is available in the original publication.^7^ To reduce bias from population stratification, we limited our investigation to a European ancestry sample, which was identified using principal component analysis in the original GWAS.^7^ Given the phenotypic and genetic heterogeneity of epilepsies, we retrieved summary statistics separately for epilepsy (all epilepsies) (n cases=27 559), focal epilepsies (n cases=14 939) and genetic generalised epilepsies (n cases=6952) (table 1) per the diagnoses of epilepsy specialists in the original GWAS.^7^

**Table 1:** The investigated phenotypes, number of cases and controls contributing to the GWAS and the number of single-nucleotide polymorphisms qualifying as instruments for Mendelian randomisation

Phenotype	Study/Consortium	N cases	N controls	Number of instruments (mean F-statistic)
Epilepsy (broadly defined)*	ILAE^7^	27 559	42 436	36–42 (25.38–25.54)
Generalised epilepsy	ILAE^7^	6952	42 436	65–80 (27.63–27.93)
Focal epilepsy	ILAE^7^	14 939	42 436	24–28 (23.06–23.15)
Anorexia nervosa	Watson *et al* 17	16 992	55 525	39 (24.33)
Anxiety disorder	Otowa *et al* 18	7016	14 745	3 (26.21)
Attention-deficit/hyperactivity disorder	Demontis *et al* 19	38 691	186 843	102 (27.19)
Autism	Grove *et al* 20	18 381	27 969	21 (24.26)
Bipolar disorder	Mullins *et al* 21	41 917	371 549	187 (27.14)
Depression	Als *et al* 22	176 143	528 770	174 (27.85)
Intelligence	Savage *et al* 23	269 867	–	487 (30.96)
OCD	IOCDF-GC and OCGAS^24^	2688	7037	7 (22.04)
Schizophrenia	Trubetskoy *et al* 25	67 390	94 015	489 (32.35)
Suicide attempts	Mullins *et al* 26	26 590	492 022	29 (24.32)
Tic-disorders (Tourette syndrome)	Yu *et al* 27	4819	9488	9 (23.81)

*For epilepsy, the number of instruments and mean F-statistic varied (past the decimal) due to individual harmonisation to outcome GWAS (a procedure employed to maximise power), reported values represent the minimum and maximum values.

GWAS, genome-wide association studies; ILAE, International League Against Epilepsy; IOCDF-GC, International Obsessive Compulsive Disorder Foundation Genetics Collaborative; OCD, obsessive-compulsive disorder; OCGAS, OCD Collaborative Genetics Association Studies.

We then extracted summary statistics from the European ancestry GWAS of commonly studied psychiatric comorbidities of epilepsy (table 1). The recent ILAE GWAS^7^ performed linkage disequilibrium score regression (LDSC) on some psychiatric phenotypes. However, we extended this analysis by using larger GWAS datasets and investigating a broader spectrum of psychiatric conditions. These included GWAS on anorexia nervosa,^17^ anxiety,^18^ attention-deficit/hyperactivity disorder,^19^ autism,^20^ bipolar disorder,^21^ depression,^22^ intelligence,^23^ obsessive-compulsive disorder,^24^ schizophrenia,^25^ suicide attempts^26^ and tic-disorders (Tourette syndrome).^27^ Detailed descriptions of the phenotypes and GWAS analyses can be found in the original publications.^17–27^

### Genetic correlations—cross-trait linkage disequilibrium score regression

To quantify the genetic correlation between epilepsy and psychiatric comorbidities, we employed cross-trait LDSC.^14 15^ LDSC leverages the pattern of linkage disequilibrium among common genetic variants to estimate the genetic correlation across phenotypes.^15^ We obtained precomputed linkage disequilibrium scores based on the 1000 Genomes Project European Reference Panel. We estimated the LDSC using an unconstrained intercept to allow for sample overlap and population stratification. Univariate LDSC criteria for consistent estimation appeared to be generally satisfied (heritability Z-score >1.5, mean χ^2^ >1.02, single-nucleotide polymorphism (SNP) heritability intercept 0.9 to 1.1).^28^ A Bonferroni correction of 11×3 test was applied for the genetic correlation analysis (α=0.001515).

### Mendelian randomisation

To assess the potential effect of epilepsy on psychiatric conditions and these conditions on epilepsy, we employed bidirectional two-sample MR. MR is based on the principles of instrumental variable analysis using common genetic variants that are robustly associated with a phenotype (exposure) of interest as instruments, yielding an estimate of the causal effect of genetic liability for a phenotype on the outcome of interest if the genetic variant is a valid instrument.^16^ A genetic variant is a valid instrument if (i) it is robustly associated with the exposure (relevance), (ii) it is not associated with any measured/unmeasured confounders of the exposure-outcome associations (independence) and (iii) it does not affect the outcome via pathways other than the potential effect on the exposure of interest (exclusion restriction).^29^ Importantly, as genetic variants are fixed at conception and randomly assorted during meiosis, MR is considered robust against reverse causality and many forms of environmental confounding factors.

In two-sample MR, SNP-exposure and SNP-outcome estimates are extracted from two independent GWASs of the same underlying population.^29^ In bidirectional MR, the procedure is repeated in both postulated directions in separate analyses.

To derive the instruments for our MR analyses, we pooled exposure and outcome GWAS data and excluded non-overlapping SNPs. To retain the highest number of SNPs, we repeated this procedure for each psychiatric GWAS separately. From these datasets, we then extracted all variants with a p value <5×10^−06^, a threshold employed to maximise power. This inclusive approach may, under some scenarios, lead to a conservative estimate in the two-sample MR because of weak instruments (so-called weak instrument bias), assuming the absence of sample overlap between GWASs. The same p value threshold has been previously employed in studies of psychiatric phenotypes.^30^ The identified variants were clumped using PLINK LD-clumping, with an R^2^ <0.01 and a 10 000 kb window, based on the 1000 Genome European phase III reference panel. SNPs from the epilepsy GWAS were then extracted from the GWAS of each outcome and harmonised to ensure that the effect estimates were expressed on the same allele.^31^

For the primary analysis, we employed the inverse-variance-weighted (IVW) method, which estimates the causal effect under the assumption that all instruments are valid.^29^ Specifically, the IVW method is the ratio of SNP-exposure and SNP-outcome associations weighted by their relative precision. Because the assumptions of IVW are strong^32^ (eg, no horizontal pleiotropy), we performed a series of standard sensitivity analyses to assess the robustness of the IVW causal effect estimates (see online supplemental material). A Bonferroni correction of 11×3×2 test was applied for the MR (α=0.00075758).

## Results

### Population-wide lifetime comorbidity

**Figure 2:**
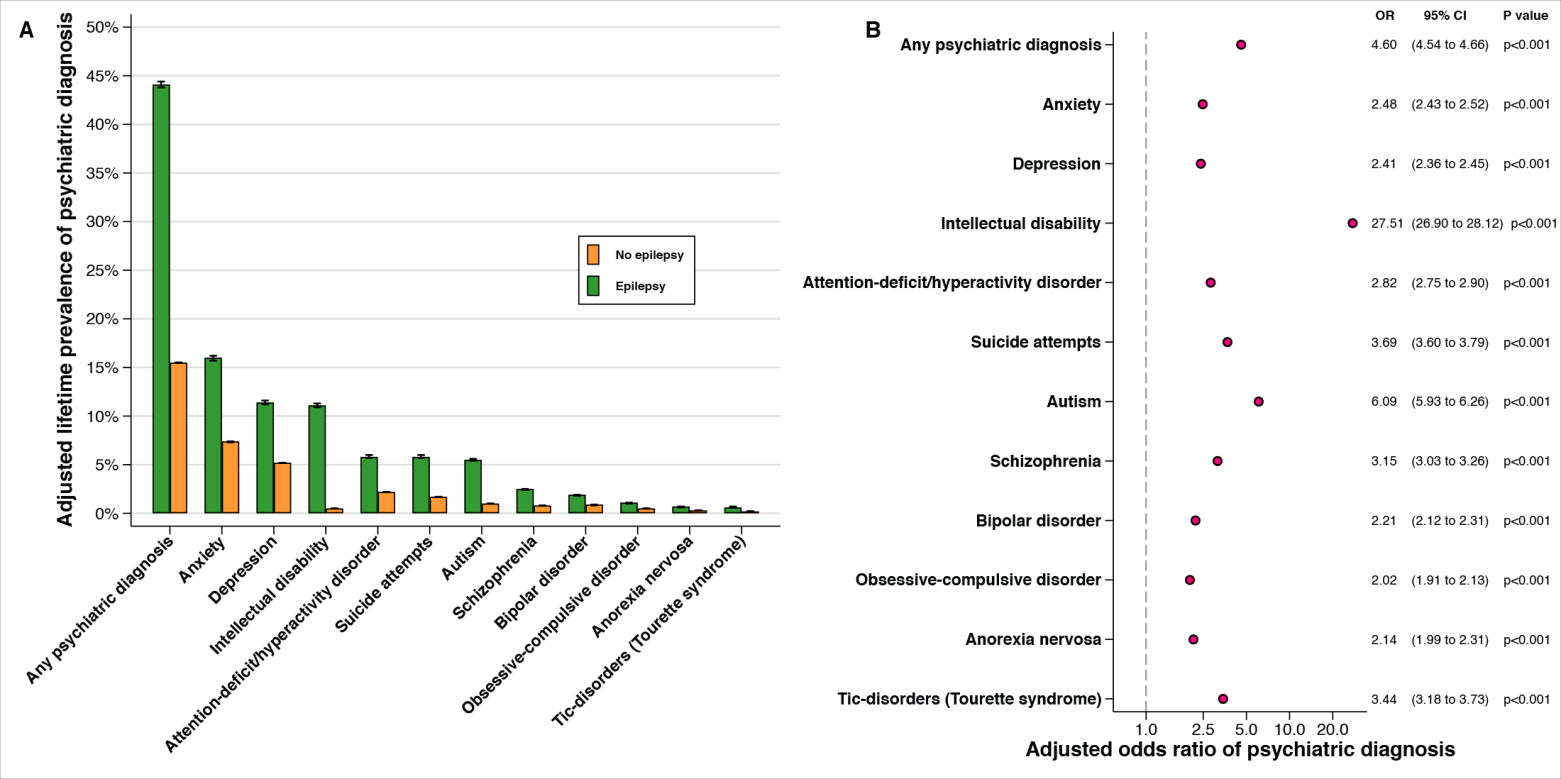
The population-wide co-occurrence of epilepsy and psychiatric morbidities. (A) The adjusted (marginal) lifetime prevalence of psychiatric diagnoses among those with (n=94 435) and without an epilepsy diagnosis (n=7 534 060). (B) The adjusted (conditional) OR of psychiatric diagnoses comparing those with and without an epilepsy diagnosis. All analyses are adjusted for birth year, sex, highest achieved disposable income and immigration status. Both panels are sorted according to the adjusted lifetime prevalence among those with an epilepsy diagnosis. Black lines indicate 95% CIs in both panels (CIs are plotted in (B) but they are smaller than the marker size, reflecting our large sample size). CI, confidence interval; OR, odds ratio.

We observed substantial psychiatric morbidity among patients with epilepsy. Almost half of all individuals diagnosed with epilepsy were also diagnosed with a (neuro-)psychiatric disorder in their lifetime (adjusted lifetime prevalence (%) 44.09, 95% CI 43.78 to 44.39; OR 4.60, 95% CI 4.54 to 4.66, p*<*0.001) (figure 2A), compared with 15.52% among those with no diagnosis of epilepsy (adjusted lifetime prevalence (%) 15.52, 95% CI 15.50 to 15.55) (for extended results, see online supplemental table 2). Each of the considered psychiatric disorders was more common among those diagnosed with epilepsy (figure 2B), than among those who were not, although the absolute lifetime prevalence of specific psychiatric disorders varied according to the underlying prevalence in the population. There was no apparent distinction between the co-occurrence of neurodevelopmental and psychiatric disorders; both forms of comorbidity were more common among those with epilepsy. The highest relative occurrence was observed for autism (OR 6.09, 95% CI 5.93 to 6.26) and intellectual disability. Intellectual disability was 27 times more common among those with epilepsy (adjusted lifetime prevalence (%) 11.12, 95% CI 10.94 to 11.30; OR 27.51, 95% CI 26.9 to 28.12), compared with those not diagnosed with epilepsy (adjusted lifetime prevalence (%) 0.51, 95% CI 0.51 to 0.52).

The estimated lifetime comorbidity did not materially differ before or after controlling for covariates, although all estimates were elevated in crude analysis (unadjusted lifetime prevalence of any psychiatric diagnosis (%) 48.61, 95% CI 48.29 to 48.93; OR 5.16, 95% CI 5.09 to 5.23, p*<*0.001) (online supplemental figure 2 and online supplemental table 2). Lifetime comorbidity did not vary materially between men and women (online supplemental figure 3) or between focal and generalised epilepsy (online supplemental figure 4) and was not limited to recent birth cohorts (online supplemental figure 5).

### Genetic correlation

**Figure 3:**
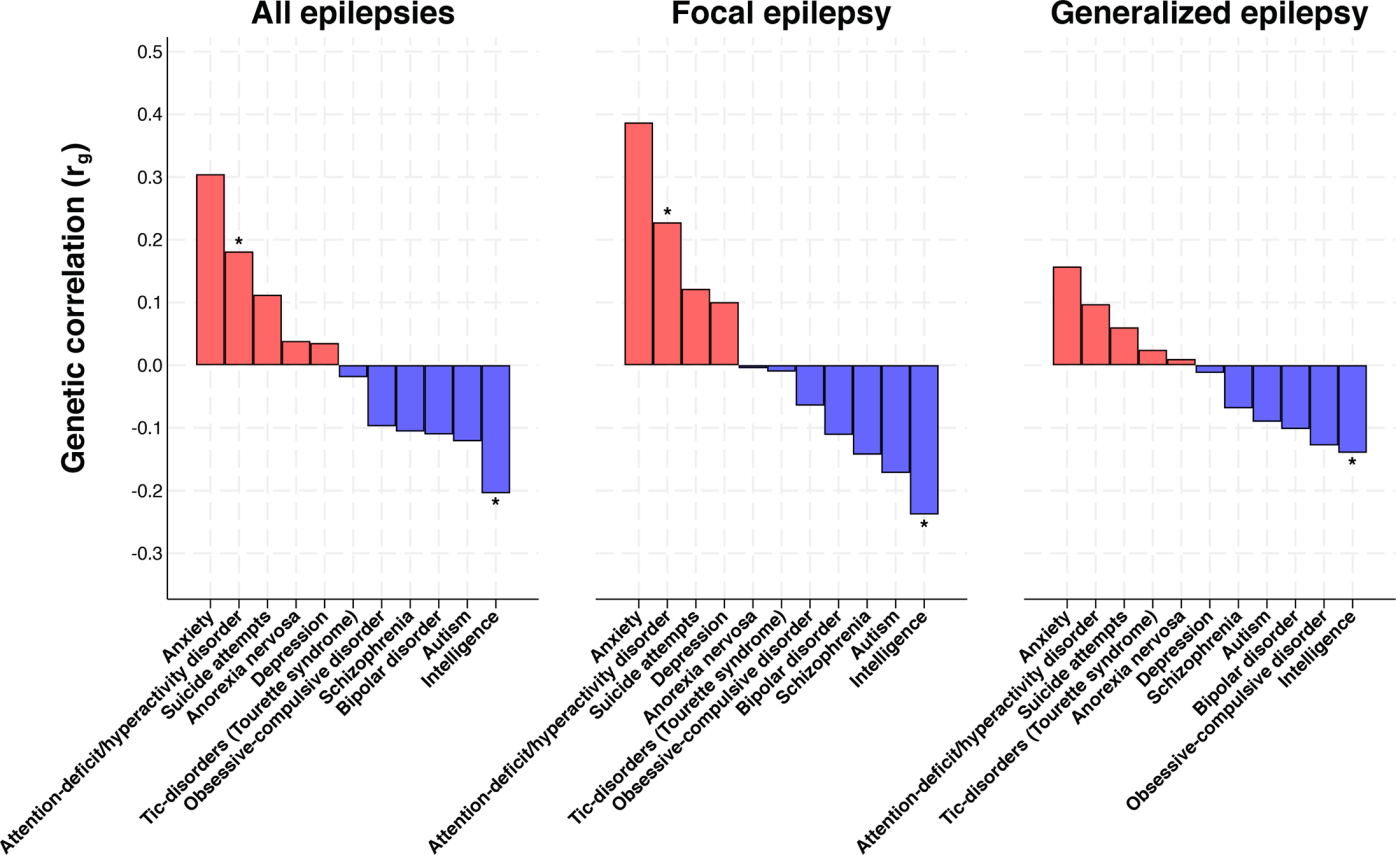
The genetic correlation between epilepsy (all epilepsies, generalised and focal) and common psychiatric comorbidities of epilepsy. Estimates represent the genetic correlation obtained from cross-trait linkage disequilibrium score regression. Asterisks reflect a p value lower than the Bonferroni-corrected threshold (α=0.001515).

We observed a positive genetic correlation between epilepsy and attention-deficit/hyperactivity disorder (r_g_=0.18, 95% CI 0.09 to 0.27, p*<*0.001) (figure 3) (for extended results see online supplemental tables 3–5). The genetic correlation between epilepsy and attention-deficit/hyperactivity disorder was more pronounced for focal epilepsy (r_g_=0.23, 95% CI 0.09 to 0.36, p*<*0.001). Furthermore, we identified a negative genetic correlation between epilepsy and intelligence (r_g_=−0.20, 95% CI −0.28 to −0.13, p*<*0.001), which was also more pronounced for focal epilepsy (r_g_=−0.24, 95% CI −0.36 to −0.12, p*<*0.001). The negative genetic correlation between epilepsy and intelligence may be consistent with the observational association between epilepsy and intellectual disability, as the intelligence scale is inversely related to intellectual disability. We observed modest-to-weak genetic correlations between the remaining psychiatric disorders and epilepsy, regardless of the type of epilepsy (figure 3).

### Mendelian randomisation

**Figure 4:**
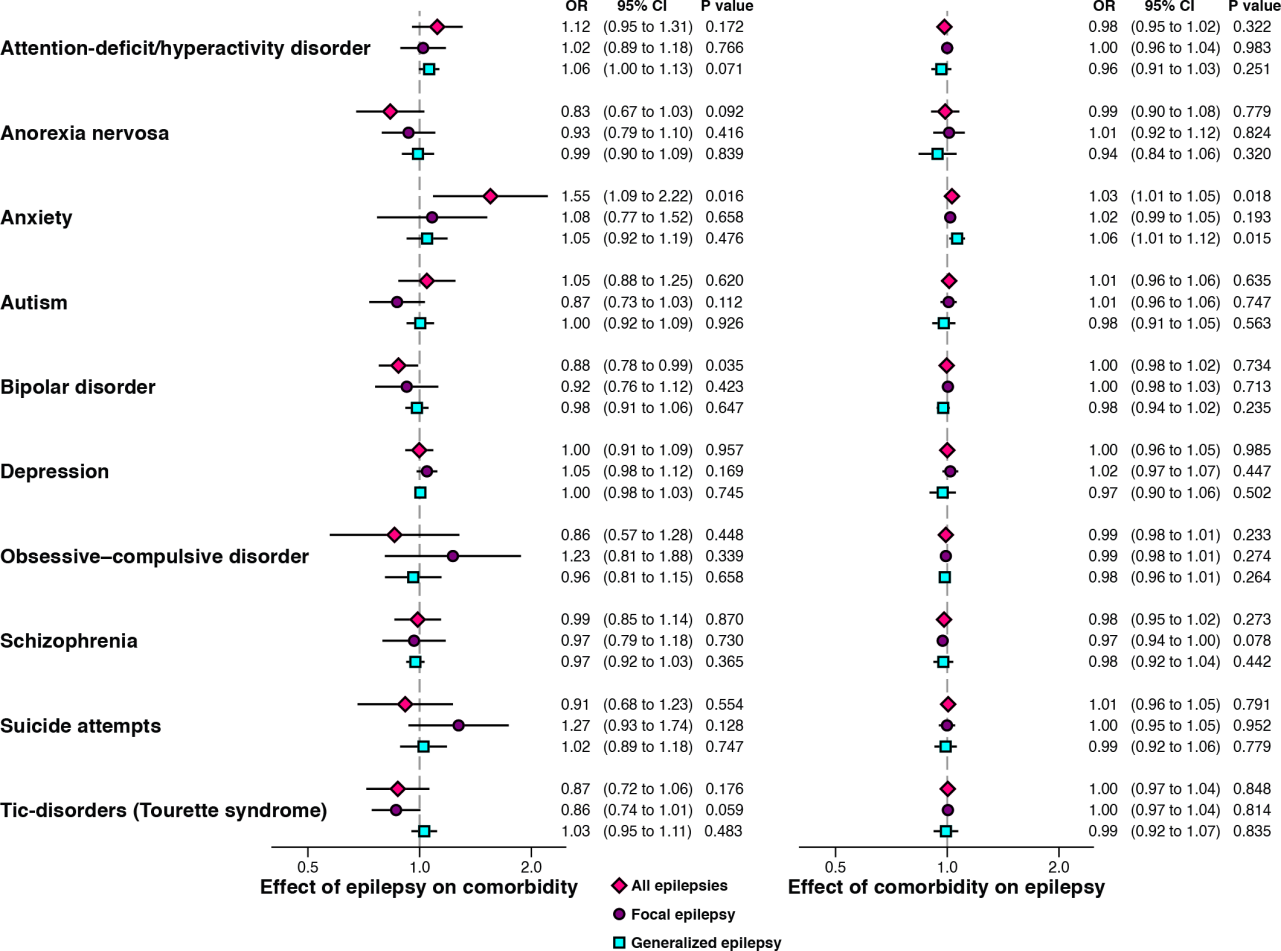
The bidirectional relationship between genetic liability to epilepsy (all epilepsies, generalised and focal) and genetic liability to common psychiatric comorbidities of epilepsy. The estimates are ORs obtained from an inverse-variance weighted regression, which assumes that all variants are valid instruments. Findings for intelligence are presented in-text, as intelligence is measured on a continuous scale and therefore not scale-comparable to the other traits. Both x-axes are plotted on the natural logarithmic scale. CI, confidence interval; OR, odds ratio.

In the MR analyses, limited evidence existed of an association between genetic liability to epilepsy and any of the considered psychiatric conditions after Bonferroni correction (figure 4) (for extended results, see online supplemental tables 3–5). The largest observed association was for genetic liability to epilepsy and anxiety disorder, which did not pass Bonferroni correction (p>0.000758). Specifically, genetic liability to epilepsy was associated with an increased likelihood of anxiety disorder (OR 1.55, 95% CI 1.09 to 2.22, p=0.016); a finding that was not replicated in sensitivity analysis (online supplemental methods and online supplemental table 7).

Limited evidence existed of an association between genetic liability to most psychiatric conditions and epilepsy (figure 4). One exception was intelligence. Genetic predisposition to higher intelligence reduced the likelihood of epilepsy (OR 0.89, 95% CI 0.85 to 0.93, p*<*0.001), and the focal (OR 0.88, 95% CI 0.84 to 0.92, p*<*0.001) and generalised subtypes of epilepsy (OR 0.87, 95% CI 0.87 to 0.94, p*<*0.001).

Although there were strong assumptions in our primary MR analysis (IVW), our findings were mostly consistent across sensitivity analyses (online supplemental table 7). Generally, our sensitivity analysis was inconsistent with widespread horizontal pleiotropy in our MR analysis (online supplemental figure 6 and online supplemental table 6). That is, the MR-Egger intercepts did not suggest that the selected genes were invalid instruments through an independent effect on the outcome (violation of the third MR assumption: exclusion restriction).

## Discussion

### Main findings

Using data from nationwide health registries in Sweden, we found that individuals with epilepsy had a substantially higher prevalence of neurodevelopmental and psychiatric comorbidities. Using cross-trait linkage disequilibrium analysis, we found genetic correlations between epilepsy and certain psychiatric conditions, which, in some cases, such as attention-deficit/hyperactivity disorder, were more pronounced in focal epilepsy. The majority of genetic correlations observed between epilepsy and psychiatric conditions were modest, supporting the partial role of a genetic basis for these comorbidities. Finally, by leveraging common genetic variations, we did not find strong evidence to suggest that these comorbidities are explained by the effects of epilepsy on psychiatric conditions or psychiatric conditions on epilepsy. Taken together, these findings suggest that there are important genetic correlations between epilepsy and some psychiatric conditions. However, these are unlikely to explain the full extent of psychiatric comorbidities in epilepsy.

Our finding that the genetic correlation between epilepsy and attention-deficit/hyperactivity disorder is more pronounced in focal epilepsy is novel, although the general correlation between epilepsy and attention-deficit/hyperactivity disorder is consistent with recent work.^9 33^ For example, a recent study on the co-aggregation of epilepsy and attention-deficit/hyperactivity disorder in Swedish families found a strikingly similar genetic correlation (0.21, 95% CI 0.02 to 0.40),^33^ although they were unable to explore subtypes of epilepsy. Our findings suggest that focal epilepsy is the main cause of this association. This contrasts with the aim of a previous study that examined generalised epilepsy because of its higher heritability.^9^ However, using updated GWAS data, we also confirmed the identification of a shared genetic overlap between epilepsy and attention-deficit/hyperactivity disorder.^9^ Although focal epilepsy has a lower heritability than generalised epilepsy,^7^ our findings suggest that pleiotropic genes are distinctly associated with focal epilepsy and attention-deficit/hyperactivity disorder. However, we noted that there was no substantial difference in the population-wide prevalence of psychiatric comorbidities between focal and generalised epilepsy (online supplemental figure 4), although subtype characterisation of epilepsy in Swedish registries may not be accurately recorded, and many individuals may have multiple seizure types.

Consistent with the population rate of intellectual disability among those with epilepsy (OR 27.5, p<0.001), we found that epilepsy and intelligence had a negative genetic correlation, and that genetic liability to higher intelligence was associated with a lower risk of epilepsy. Our findings are consistent with a shared genetic aetiology of intellectual disability and epilepsy. However, it is important to acknowledge that intelligence and intellectual disability are two distinct phenotypes. We were only able to perform MR and genetic correlation analyses of intelligence because of the absence of GWAS data for intellectual disabilities.

Intriguingly, using MR, we were unable to confirm the common assumption that epilepsy is a ‘cause’ of an increased risk of psychiatric disorders, or that these disorders are a ‘cause’ for an increased risk of epilepsy.^12^ This finding should be interpreted in light of the MR assumptions being met and the possibility of the role of rare genetic variations not captured within the GWAS data which comprise common variants.

However, this finding may also indicate the importance of environmental factors in the origin of psychiatric comorbidities in epilepsy. Future work could help identify the causal role of such factors (eg, prenatal or perinatal events unique to the individual, or discrimination, stigma and life events during the individual’s lifetime, which are common risk factors for several neurological and psychiatric phenotypes^2 33 34^), ideally using causal triangulation across study designs with orthogonal sources of bias.

### Limitations

An important limitation to note is that existing epilepsy and psychiatric conditions GWAS are not sufficiently large, potentially leading to reduced power in identifying genetic correlations, and necessitating an inclusive threshold (p<5×10^−6^) to identify instruments for MR analyses. Although this threshold has been widely employed in previous studies, the possibility of reduced specificity in instrument selection, leading to weak instrument bias, cannot be ruled out. Analyses like ours, repeated using a larger GWAS, when available, may provide stronger evidence. Furthermore, rare genetic variants are known to play an important role in the aetiology of epilepsy,^7^ and their contribution to psychiatric comorbidities in epilepsy cannot be excluded. Although rare genetic variants may not be sufficiently common in the population to explain the high rates of psychiatric conditions reported in observational studies including our nationwide study, they may still be common among individuals with epilepsy. Nonetheless, future studies will help clarify the role of rare genetic variants in the overlap between psychiatric conditions and epilepsy. We were also unable to perform any age-stratified or sex-stratified cross-trait genetic analysis; however, this could be a target for future analysis if sex-stratified GWAS data were available. Another limitation of the Swedish data analysis is that our study relied on the accuracy of the epilepsy and psychiatric diagnoses recorded in the registers. Validation studies of epilepsy^35^ and a range of psychiatric and neurodevelopmental conditions suggest a high validity of these recorded diagnoses. However, the possibility of measurement bias in the data cannot be excluded. This limitation is common across large-scale epidemiological studies. Further large-scale efforts to estimate the burden of psychiatric conditions among people with epilepsy are warranted, with particular emphasis on including diverse and heterogeneous populations. Future work based on high-resolution phenotyping of smaller representative cohorts will shed further light on the complexities of the relationships studied.

### Implications

Our study confirms the notable prevalence of psychiatric comorbidities among individuals with epilepsy and provides mechanistic insights into this co-occurrence. These findings underscore the importance of comprehensive patient care that acknowledges and addresses associated conditions during epilepsy diagnosis and treatment. This is particularly important, as psychiatric comorbidities notably impact the prognosis of epilepsy, influencing mortality and life expectancy.^36 37^ While our study revealed some genetic correlations suggesting a potential genetic contribution to comorbidities, it did not strongly support bidirectional relationships. Consequently, our study emphasises the importance of exploring alternative factors, including environmental influences and rare genetic variations, to unravel the origins of psychiatric comorbidities in epilepsy. Understanding the underlying causes of comorbidities is crucial, as it can guide treatment decisions and offer more effective strategies for managing both epilepsy and associated psychiatric conditions. This may enhance the overall quality of care for individuals affected by epilepsy.

In conclusion, psychiatric comorbidities are common in patients with epilepsy. Genetic correlations may partly explain some comorbidities; however, little evidence exists of a bidirectional relationship between the genetic liability of epilepsy and psychiatric conditions. These findings highlight the need to understand the role of environmental factors or rare genetic variations in the origins of psychiatric comorbidities in epilepsy.

## Data Availability

No data are available. All genetic data produced in this study are publicly available in their original publication and upon reasonable request from the authors. Swedish privacy laws prohibit the authors from making registry data publicly available. The data supporting these findings were used under licence and ethical approval for this study. Readers interested in obtaining microdata or replicating this study may seek similar approval and enquiries from Statistics Sweden. For further advice, see https://www.scb.se/en/services/guidance-for-researchers-and-universities/, or contact Statistics Sweden at mikrodata@scb.se.

## References

[1] Fazel S , Wolf A , Långström N , et al . Premature mortality in epilepsy and the role of psychiatric comorbidity: a total population study. Lancet 2013;382:1646–54. 10.1016/S0140-6736(13)60899-5 23883699 PMC3899026

[2] Keezer MR , Sisodiya SM , Sander JW . Comorbidities of epilepsy: current concepts and future perspectives. Lancet Neurol 2016;15:106–15. 10.1016/S1474-4422(15)00225-2 26549780

[3] Shimizu H , Morimoto Y , Yamamoto N , et al . Overlap between epilepsy and neurodevelopmental disorders: insights from clinical and genetic studies. In: Czuczwar SJ , ed. Epilepsy. Brisbane (AU): Exon Publications, 2022: 41–54. 35605085

[4] Chow J , Jensen M , Amini H , et al . Dissecting the genetic basis of comorbid epilepsy phenotypes in neurodevelopmental disorders. Genome Med 2019;11:65. 10.1186/s13073-019-0678-y 31653223 PMC6815046

[5] Tellez-Zenteno JF , Patten SB , Jetté N , et al . Psychiatric comorbidity in epilepsy: a population-based analysis. Epilepsia 2007;48:2336–44. 10.1111/j.1528-1167.2007.01222.x 17662062

[6] Rai D , Kerr MP , McManus S , et al . Epilepsy and psychiatric comorbidity: a nationally representative population-based study. Epilepsia 2012;53:1095–103. 10.1111/j.1528-1167.2012.03500.x 22578079

[7] International League Against Epilepsy Consortium on Complex Epilepsies . GWAS meta-analysis of over 29,000 people with epilepsy identifies 26 risk Loci and subtype-specific genetic architecture. Nat Genet 2023;55:1471–82. 10.1038/s41588-023-01485-w 37653029 PMC10484785

[8] Campbell C , Cavalleri GL , Delanty N . Exploring the genetic overlap between psychiatric illness and epilepsy: a review. Epilepsy Behav 2020;102:106669. 10.1016/j.yebeh.2019.106669 31785486

[9] Wu Y , Li Y , Zhu J , et al . Shared genetics and causality underlying epilepsy and attention-deficit hyperactivity disorder. Psychiatry Res 2022;316:114794. 10.1016/j.psychres.2022.114794 35994864

[10] Yuan S , Tomson T , Larsson SC . Modifiable risk factors for epilepsy: a two-sample Mendelian randomization study. Brain Behav 2021;11:e02098. 10.1002/brb3.2098 33655641 PMC8119863

[11] Li G , Wang M , Zheng M , et al . Causal effect of psychiatric disorders on epilepsy: A two-sample Mendelian randomization study. Brain Behav 2023;13:e2939. 10.1002/brb3.2939 36860142 PMC10097067

[12] Kanner AM , Ribot R , Mazarati A . Bidirectional relations among common psychiatric and neurologic comorbidities and epilepsy: do they have an impact on the course of the seizure disorder. Epilepsia Open 2018;3:210–9. 10.1002/epi4.12278 30564780 PMC6293067

[13] Kanner AM . Can neurobiological pathogenic mechanisms of depression facilitate the development of seizure disorders. Lancet Neurol 2012;11:1093–102. 10.1016/S1474-4422(12)70201-6 23021976

[14] Bulik-Sullivan B , Finucane HK , Anttila V , et al . An atlas of genetic correlations across human diseases and traits. Nat Genet 2015;47:1236–41. 10.1038/ng.3406 26414676 PMC4797329

[15] Bulik-Sullivan BK , Loh P-R , Finucane HK , et al . LD score regression distinguishes confounding from polygenicity in genome-wide association studies. Nat Genet 2015;47:291–5. 10.1038/ng.3211 25642630 PMC4495769

[16] Smith GD , Ebrahim S . 'Mendelian randomization': can genetic epidemiology contribute to understanding environmental determinants of disease Int J Epidemiol 2003;32:1–22. 10.1093/ije/dyg070 12689998

[17] Watson HJ , Yilmaz Z , Thornton LM , et al . Genome-wide association study identifies eight risk Loci and implicates metabo-psychiatric origins for anorexia nervosa. Nat Genet 2019;51:1207–14. 10.1038/s41588-019-0439-2 31308545 PMC6779477

[18] Otowa T , Hek K , Lee M , et al . Meta-analysis of genome-wide association studies of anxiety disorders. Mol Psychiatry 2016;21:1391–9. 10.1038/mp.2015.197 26754954 PMC4940340

[19] Demontis D , Walters GB , Athanasiadis G , et al . Genome-wide analyses of ADHD identify 27 risk loci, refine the genetic architecture and implicate several cognitive domains. Nat Genet 2023;55:198–208. 10.1038/s41588-022-01285-8 36702997 PMC10914347

[20] Grove J , Ripke S , Als TD , et al . Identification of common genetic risk variants for autism spectrum disorder. Nat Genet 2019;51:431–44. 10.1038/s41588-019-0344-8 30804558 PMC6454898

[21] Mullins N , Forstner AJ , O’Connell KS , et al . Genome-wide association study of more than 40,000 bipolar disorder cases provides new insights into the underlying biology. Nat Genet 2021;53:817–29. 10.1038/s41588-021-00857-4 34002096 PMC8192451

[22] Als TD , Kurki MI , Grove J , et al . Depression pathophysiology, risk prediction of recurrence and comorbid psychiatric disorders using genome-wide analyses. Nat Med 2023;29:1832–44. 10.1038/s41591-023-02352-1 37464041 PMC10839245

[23] Savage JE , Jansen PR , Stringer S , et al . Genome-wide association meta-analysis in 269,867 individuals identifies new genetic and functional links to intelligence. Nat Genet 2018;50:912–9. 10.1038/s41588-018-0152-6 29942086 PMC6411041

[24] International Obsessive Compulsive Disorder Foundation Genetics Collaborative (IOCDF-GC) and OCD Collaborative Genetics Association Studies (OCGAS) . Revealing the complex genetic architecture of obsessive-compulsive disorder using meta-analysis. Mol Psychiatry 2018;23:1181–8. 10.1038/mp.2017.154 28761083 PMC6660151

[25] Trubetskoy V , Pardiñas AF , Qi T , et al . Mapping genomic loci implicates genes and synaptic biology in schizophrenia. Nature 2022;604:502–8. 10.1038/s41586-022-04434-5 35396580 PMC9392466

[26] Mullins N , Kang J , Campos AI , et al . Dissecting the shared genetic architecture of suicide attempt, psychiatric disorders, and known risk factors. Biol Psychiatry 2022;91:313–27. 10.1016/j.biopsych.2021.05.029 34861974 PMC8851871

[27] Yu D , Sul JH , Tsetsos F , et al . Interrogating the Genetic Determinants of Tourette's Syndrome and Other Tic Disorders Through Genome-Wide Association Studies. Am J Psychiatry 2019;176:217–27. 10.1176/appi.ajp.2018.18070857 30818990 PMC6677250

[28] Zheng J , Erzurumluoglu AM , Elsworth BL , et al . LD Hub: a centralized database and web interface to perform LD score regression that maximizes the potential of summary level GWAS data for SNP heritability and genetic correlation analysis. Bioinformatics 2017;33:272–9. 10.1093/bioinformatics/btw613 27663502 PMC5542030

[29] Burgess S , Butterworth A , Thompson SG . Mendelian randomization analysis with multiple genetic variants using summarized data. Genet Epidemiol 2013;37:658–65. 10.1002/gepi.21758 24114802 PMC4377079

[30] Choi KW , Chen C-Y , Stein MB , et al . Assessment of Bidirectional Relationships Between Physical Activity and Depression Among Adults: A 2-Sample Mendelian Randomization Study. JAMA Psychiatry 2019;76:399–408. 10.1001/jamapsychiatry.2018.4175 30673066 PMC6450288

[31] Hemani G , Zheng J , Elsworth B , et al . The MR-base platform supports systematic causal inference across the human phenome. Elife 2018;7:e34408. 10.7554/eLife.34408 29846171 PMC5976434

[32] Carter AR , Fraser A , Howe LD , et al . Why caution should be applied when interpreting and promoting findings from Mendelian randomisation studies. Gen Psychiatr 2023;36:e101047. 10.1136/gpsych-2023-101047 37583791 PMC10423826

[33] Brikell I , Ghirardi L , D’Onofrio BM , et al . Familial Liability to Epilepsy and Attention-Deficit/Hyperactivity Disorder: A Nationwide Cohort Study. Biol Psychiatry 2018;83:173–80. 10.1016/j.biopsych.2017.08.006 28950988 PMC5723535

[34] Gu W , Zhao Q , Yuan C , et al . Impact of adverse childhood experiences on the symptom severity of different mental disorders: a cross-diagnostic study. Gen Psychiatr 2022;35:e100741. 10.1136/gpsych-2021-100741 35572774 PMC9036421

[35] Sveinsson O , Andersson T , Carlsson S , et al . The incidence of SUDEP: A nationwide population-based cohort study. Neurology 2017;89:170–7. 10.1212/WNL.0000000000004094 28592455

[36] Dreier JW , Laursen TM , Tomson T , et al . Cause-specific mortality and life years lost in people with epilepsy: a Danish cohort study. Brain 2023;146:124–34. 10.1093/brain/awac042 35234848

[37] Moon HJ , Lee H , Yoon D , et al . Premature Mortality and Causes of Death Among People With Epilepsy: A Nationwide Population-Based Incident Cohort Study. Neurology 2023;100:e2060–70. 10.1212/WNL.0000000000207212 36948594 PMC10186245

