# Supplementary material for: Psychiatric comorbidities in epilepsy: population co-occurrence, genetic correlations and causal effects
Source: Gen Psychiatr. 2024 Jan 30;37(1):e101201. doi: 10.1136/gpsych-2023-101201 (PMC11369844; doi:10.1136/gpsych-2023-101201)
Supplement: Supplementary data [file gpsych-2023-101201supp001.pdf]

Supplement

Supplemental Figure 1. Overview of the methodology 2

Supplemental Table 1. International Classification of Diseases codes. 3

Extended observational results 4

Supplemental Figure 2. The unadjusted population-wide co-occurrence. 5

Supplemental Figure 3. The sex-stratified population-wide co-occurrence. 6

Supplemental Figure 4. The population-wide co-occurrence stratified by type of epilepsy. 7

Supplemental Figure 5. The population-wide co-occurrence stratified by birth cohorts. 8

Extended genomic results 9

Supplemental Table 3. Extended results from the LDSC and MR analyses of epilepsy (broadly classified). 9

Supplemental Table 4. Extended results from the LDSC and MR analyses of focal epilepsy. 10

Supplemental Table 5. Extended results from the LDSC and MR analyses of generalized epilepsy. 11

eMethods - Mendelian sensitivity analysis 12

Supplemental Figure 6. Volcano plot of the MR-Egger intercept. 13

Supplemental Table 6. Egger intercepts for all Mendelian analyses. 14

Supplemental Table 7. Mendelian sensitivity analyses. 15

Supplement References 18

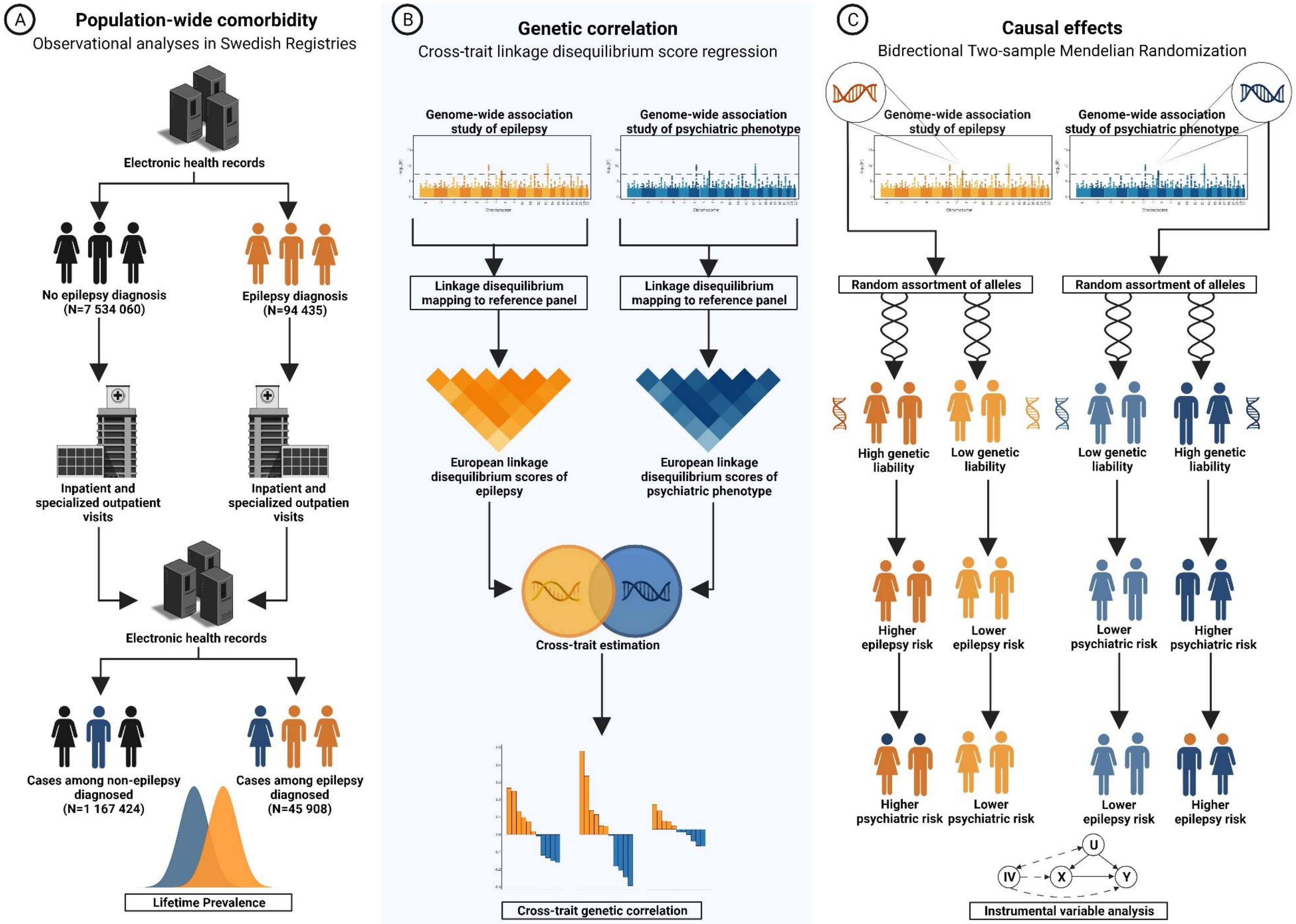

Supplemental Figure 1. Overview of the methodology employed to characterize the comorbidities of psychiatric conditions in individuals with epilepsy (A), outline the genetic correlation between epilepsy and psychiatric conditions (B) and examine the possibility of bidirectional relationship between epilepsy and psychiatric conditions (C). For a detailed description of the approaches and related assumptions see the Method section.

Supplemental Table 1. International Classification of Diseases codes used to identify conditions in the Swedish health registries.

Condition	ICD-10	ICD-9	ICD-8
Any psychiatric diagnosis	F00-F99	290-319	290-319
Attention-deficit/hyperactivity disorder	F90	314	308.3
Anorexia Nervosa	F50.0, F50.1	307.1	306.5
Anxiety Disorder	F40-F48	300.0, 300.2, 308, 309	300.0-300.2, 300.5-300.9
Autism	F84	299	299.99
Bipolar disorder	F30, F31, F34.0, F25.0	296.0, 296.2, 296.4, 296.8, 296.9	296.1, 296.3, 296.8
Depression	F32-F39	296.1, 298.0, 300.4, 311	298.0, 300.4
Epilepsy[1,2]	G40	345J-345P, 345W, 345X	345.0-345-3, 345.9
Focal	G40.0, G40.1, G40.2	345M, 345N	345.30, 345.31, 345.38, 345.39
Generalized	G40.3	345J, 345K	345.00, 345.09, 345.10, 345.11
Other and unspecified	G40.4, G40.5, G40.6, G40.7, G40.8, G40.9	345L, 345P, 345W, 345X	345.18, 345.19, 345.39, 345.32, 345.33, 345.9
Intellectual Disability	F70-F79	317-319	310-315
Obsessive-compulsive disorder	F42	300.3	300.3, 301.4
Schizophrenia	F20-F29	295.0, 295.4, 295.6, 295.8, 295.9	295.0, 295.4, 295.6, 295.8, 295.9
Suicide attempts	X60-X84	950-958	950-958
Tic-disorders (Tourette Syndrome)	F95	307.2	306.2

[1]Individuals who only received a three-character ICD code were classified as a distinct category in subtype analysis (never characterized).
[2]We were unable to classify 259 individuals who were discharged with both focal and generalized ICD codes on their most recent visit, these were retained these individuals in the "never characterized" group.

BMJ Publishing Group Limited (BMJ) disclaims all liability and responsibility arising from any reliance placed on this supplemental material which has been supplied by the author(s)

Extended observational results

Supplemental Table 2. Observational analysis[1] in Swedish health-care registries of epilepsy and psychiatric conditions.

	No Epilepsy (N=7 534 060)		Epilepsy (N=94 435)					
**Phenotype**	**N cases**	**Prevalence[2] (%) (95% CI)**	**N cases**	**Crude prevalence (%) (95% CI)**	**Adjusted prevalence (%) (95% CI)**	**Crude Odds ratio (95% CI)**	**Adjusted Odds ratio (95% CI)**	**Percent attenuation[3] (95% CI)**
Any psychiatric diagnosis	1 167 424	15.5 (15.5-15.5)	45 908	48.6 (48.3-48.9)	44.1 (43.8-44.4)	5.16 (5.09-5.23)	4.60 (4.54-4.66)	12.2 (11.8-12.5)
Anxiety	554 058	7.4 (7.4-7.4)	17 276	18.3 (18-18.5)	16 (15.7-16.2)	2.82 (2.77-2.87)	2.48 (2.43-2.52)	13.9 (13.5-14.4)
Depression	390 493	5.2 (5.2-5.2)	12 642	13.4 (13.2-13.6)	11.4 (11.2-11.6)	2.83 (2.77-2.88)	2.41 (2.36-2.45)	17.4 (17-17.9)
Intellectual disability	38 376	0.5 (0.5-0.5)	13 471	14.3 (14-14.5)	11.1 (10.9-11.3)	32.5 (31.83-33.18)	27.51 (26.9-28.12)	18.2 (17.1-19.2)
Attention-deficit/hyperactivity disorder	168 012	2.2 (2.2-2.2)	6 455	6.8 (6.7-7)	5.8 (5.7-6)	3.22 (3.13-3.3)	2.82 (2.75-2.90)	14.1 (13.3-14.9)
Suicide attempts	126 801	1.7 (1.7-1.7)	6 729	7.1 (7-7.3)	5.8 (5.7-6)	4.48 (4.37-4.6)	3.69 (3.70-3.79)	21.5 (20.9-22.1)
Autism	75 575	1.0 (1.0-1.0)	6 743	7.1 (7-7.3)	5.5 (5.4-5.6)	7.59 (7.4-7.79)	6.09 (5.93-6.26)	24.5 (23.5-25.5)
Schizophrenia	60 753	0.8 (0.8-0.8)	3 370	3.6 (3.5-3.7)	2.5 (2.4-2.5)	4.55 (4.39-4.72)	3.15 (3.03-3.26)	44.7 (43.5-46)
Bipolar disorder	64 165	0.9 (0.8-0.9)	2 287	2.4 (2.3-2.5)	1.9 (1.8-1.9)	2.89 (2.77-3.01)	2.21 (2.12-2.31)	30.7 (29.8-31.5)
Obsessive-compulsive disorder	40 491	0.5 (0.5-0.5)	1 335	1.4 (1.3-1.5)	1.1 (1-1.1)	2.65 (2.51-2.8)	2.02 (1.91-2.13)	31.4 (30.4-32.4)
Anorexia Nervosa	23 254	0.3 (0.3-0.3)	725	0.8 (0.7-0.8)	0.7 (0.6-0.7)	2.5 (2.32-2.69)	2.14 (1.99-2.31)	16.7 (15.5-18)
Tic-disorders (Tourette Syndrome)	13 874	0.2 (0.2-0.2)	654	0.7 (0.6-0.7)	0.6 (0.6-0.7)	3.78 (3.49-4.09)	3.44 (3.18-3.73)	9.8 (8.5-11.1)

[2]Estimates are obtained from logistic regression(s). Where indicated, estimates are adjusted for birth year, sex, highest achieved disposable income, and immigration status.

[2]We omit the adjusted prevalence among those without epilepsy as it did not differ before the first decimal.

[3]The percent of attenuation comparing the crude odds ratio to the adjusted odds ratio.

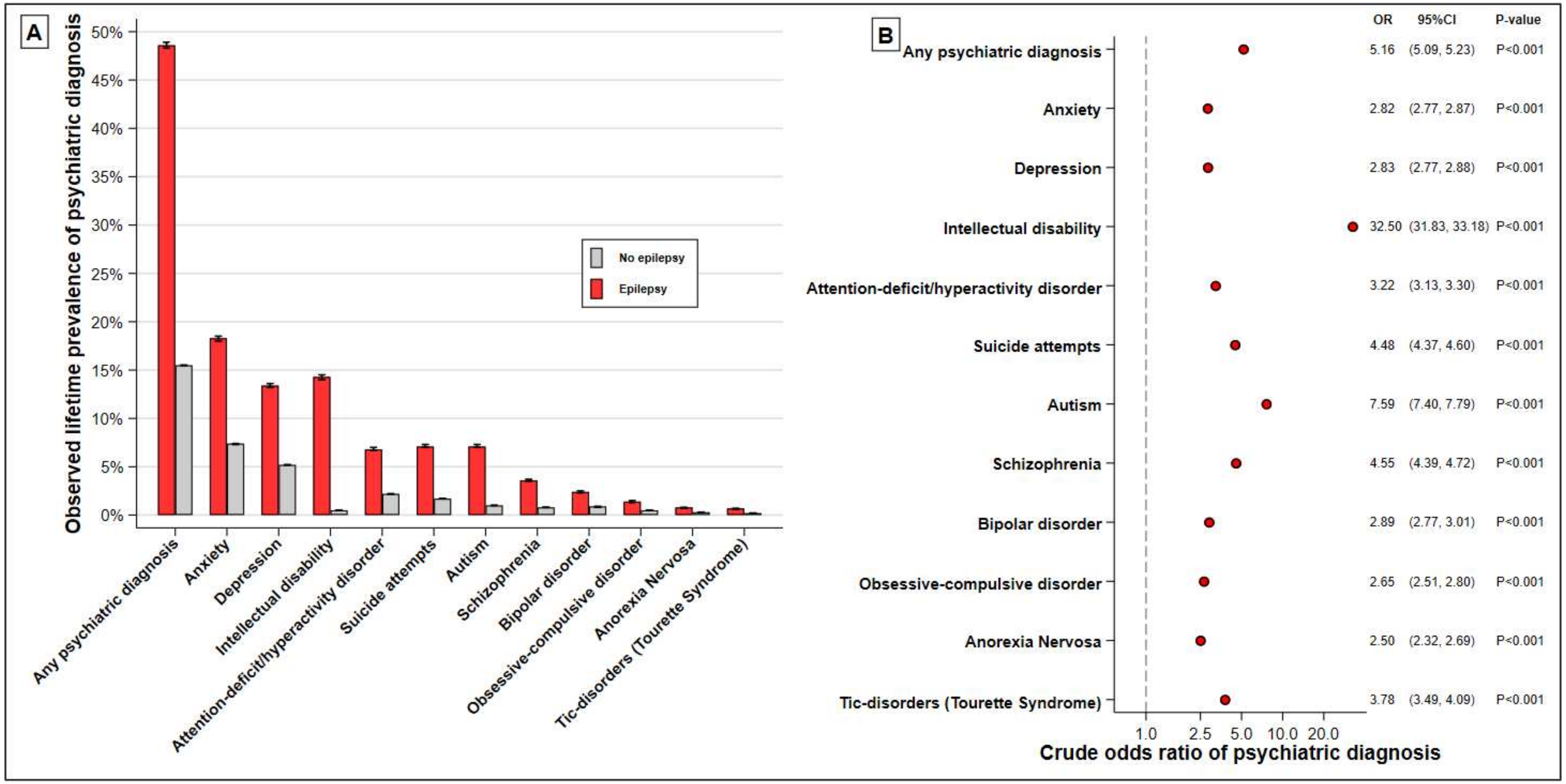

Supplemental Figure 2. The unadjusted population-wide co-occurrence of epilepsy and psychiatric morbidities. Panel A illustrates the observed lifetime prevalence of psychiatric diagnoses among those with (N=94 435) and without a diagnosis of epilepsy (N=7 534 060). Panel B illustrates the crude odds ratio of psychiatric diagnoses comparing those with and without a diagnosis of epilepsy. Black lines indicate 95% confidence intervals in both panels (confidence intervals are plotted in panel B but they are smaller than the marker size, reflecting our large sample size).

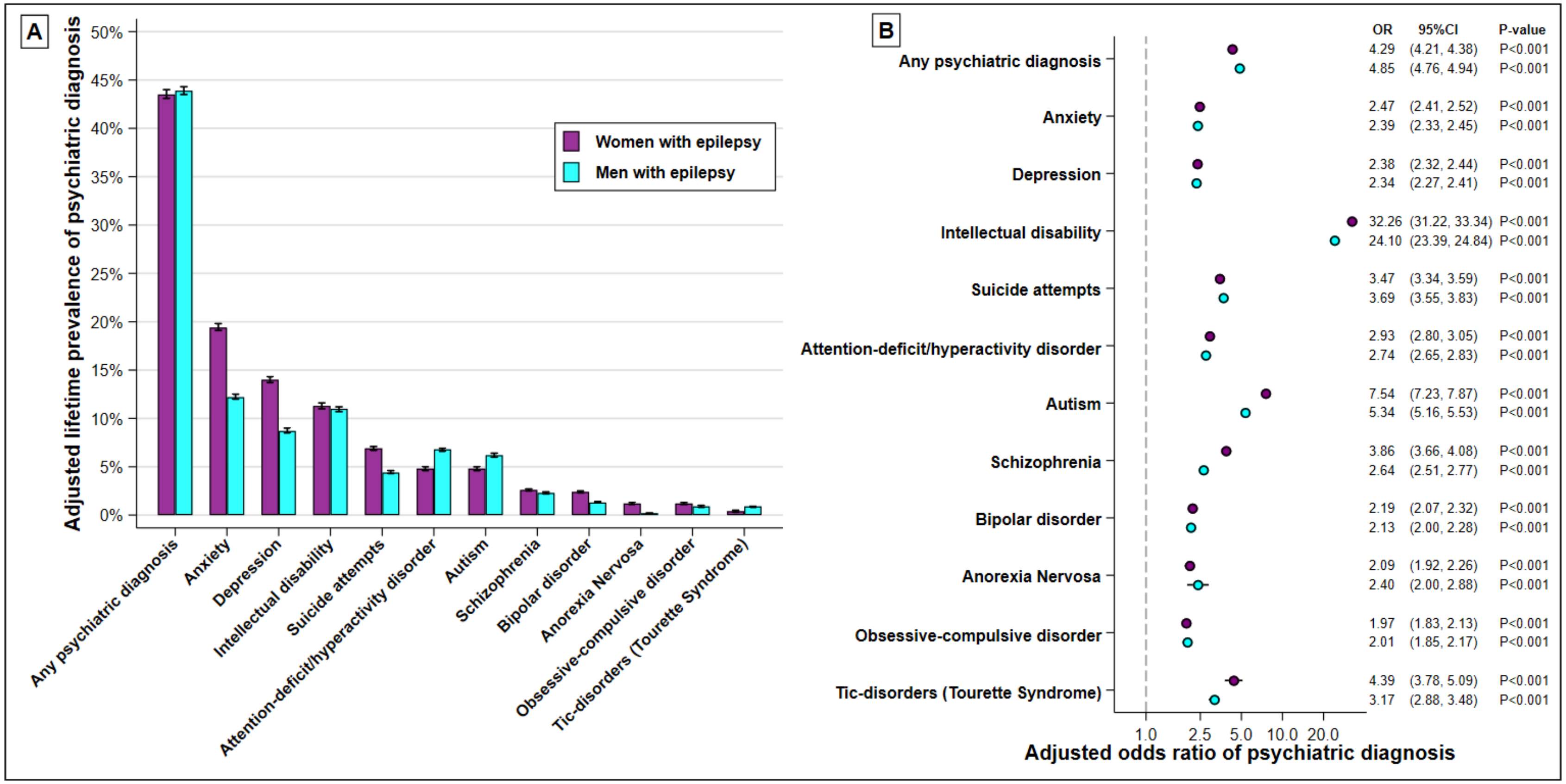

Supplemental Figure 3. The sex-stratified population-wide co-occurrence of epilepsy and psychiatric morbidities. Panel A illustrates the adjusted (marginal) lifetime probability of psychiatric diagnoses among those with a diagnosis of epilepsy (N=94 435), stratified by birth sex (purple indicating among women with epilepsy N=44 367, and cyan indicating among men with epilepsy N=50 068). Panel B illustrates the adjusted odds ratio of psychiatric diagnoses comparing those with and without a diagnosis of epilepsy, stratified by birth sex (purple indicating among women and cyan indicating among men). All analyses are adjusted for birth year, highest achieved disposable income, and immigration status. Black lines indicate 95% confidence intervals in both panels (width is sometimes smaller than marker size in panel B).

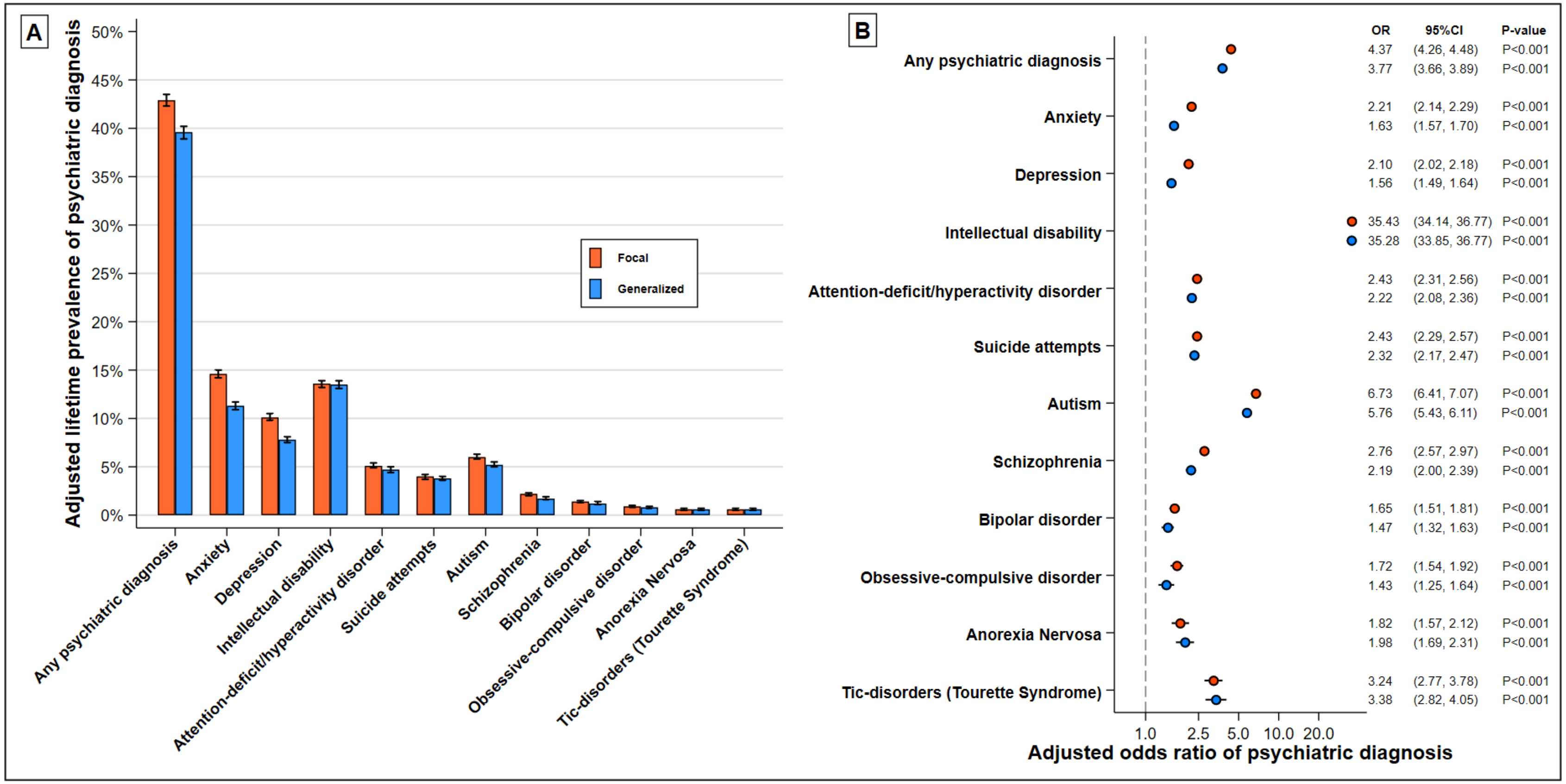

Supplemental Figure 4. The population-wide co-occurrence of epilepsy and psychiatric morbidities stratified by type of epilepsy. Panel A illustrates the adjusted (marginal) lifetime probability of psychiatric diagnoses among those with focal (N=24 430) or generalized epilepsy (N=19 199). Panel B illustrates the adjusted odds ratio of psychiatric diagnoses comparing those with focal and generalized epilepsy to those with no epilepsy diagnosis. All analyses are adjusted for birth year, highest achieved disposable income, sex, and immigration status. Black lines indicate 95% confidence intervals in both panels (width is sometimes smaller than marker size in panel B). For graphical purposes, we omit those who were never characterized (N=18 338) or other/unspecific forms of epilepsy (N=32 468) – these individuals did not differ materially in their rate of psychiatric diagnosis as compared to those with focal or generalized epilepsy.

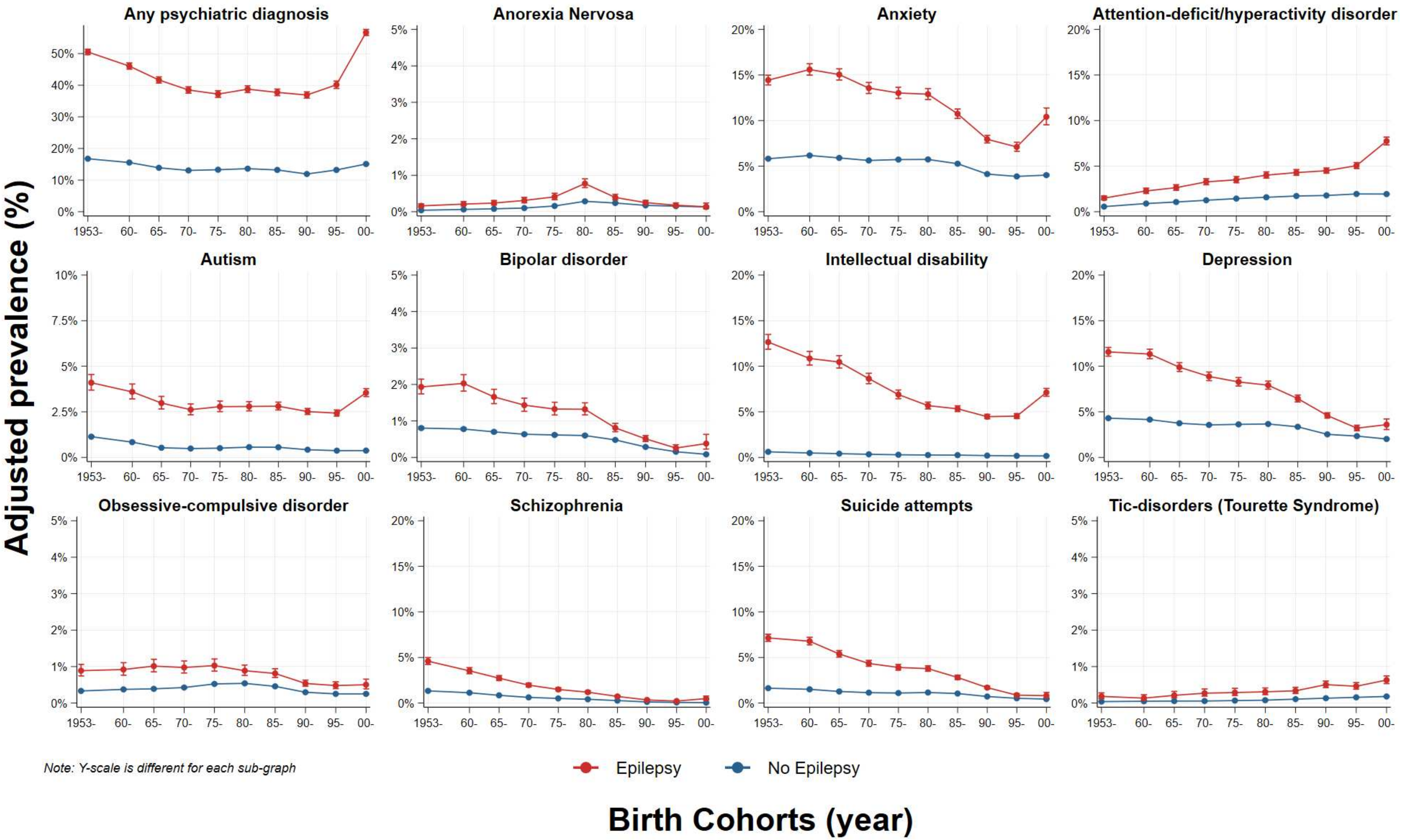

Supplemental Figure 5. The population-wide co-occurrence of epilepsy and psychiatric morbidities stratified by birth cohorts. The vertical lines illustrate the adjusted (marginal) prevalence of psychiatric diagnoses among those with (N=94 435) and without a diagnosis of epilepsy (N=7 534 060). Horizontal lines indicate 95% confidence intervals.

Extended genomic results

Supplemental Table 3. Extended results from the LDSC and MR analyses of epilepsy (broadly classified) and selected psychiatric phenotypes.

	Effect of epilepsy on comorbidity				Effect of Comorbidity on Epilepsy				Genetic correlation	
**Phenotype**	**N_{iv}**	**β**	**OR**	**P-value**	**N_{iv}**	**β**	**OR**	**P-value**	**rg**	**P-value**
Focal epilepsy	N/A	-	-	-	N/A	-	-	-	0.86	<0.001*
Generalized epilepsy	N/A	-	-	-	N/A	-	-	-	0.92	<0.001*
Anorexia Nervosa	37	-0.18	0.83	0.092	39	-0.02	0.98	0.322	0.04	0.463
Anxiety (Case-control)	39	0.44	1.55	0.016	3	-0.01	0.99	0.779	0.30	0.016
Anxiety (Continuous)[1]	39	0.05	-	0.086	6	0.01	1.01	0.897	0.06	0.638
Attention-deficit/hyperactivity disorder	37	0.11	1.12	0.172	102	0.03	1.03	0.018	0.18	<0.001*
Autism	37	0.04	1.05	0.620	21	0.01	1.01	0.635	-0.12	0.059
Bipolar disorder	37	-0.13	0.88	0.035	187	0.00	1.00	0.734	-0.11	0.005
Intelligence	42	-0.03	0.97	0.384	487	-0.12	0.89	<0.001*	-0.20	<0.001*
Depression	36	0.00	-	0.957	174	0.00	1.00	0.985	0.04	0.412
Obsessive-compulsive disorder	37	-0.16	0.86	0.448	7	-0.02	0.98	0.273	-0.10	0.244
Schizophrenia	37	-0.14	0.87	0.176	489	-0.01	0.99	0.233	-0.11	0.004
Suicide attempts	36	-0.01	0.99	0.870	29	0.01	1.01	0.791	0.11	0.070
Tic-disorders (Tourette Syndrome)	37	-0.09	0.91	0.554	9	0.00	1.00	0.848	-0.02	0.799

[1]In our primary analysis, we leverage the case-control anxiety definitions, to ensure that the findings reflect manifested anxiety. However, we chose to also analyse anxiety as a continuous score obtained from the original GWAS to enhance statistical efficiency.

*Statistically significant at Bonferroni correction (α=0.00075758 for MR and α=0.001515 for LDSC).

Supplemental Table 4. Extended results from the LDSC and MR analyses of focal epilepsy and selected psychiatric phenotypes.

	Effect of epilepsy on comorbidity				Effect of Comorbidity on Epilepsy				Genetic correlation	
**Phenotype**	**N_{iv}**	**β**	**OR**	**P-value**	**N_{iv}**	**β**	**OR**	**P-value**	**rg**	**P-value**
Epilepsy (broadly defined)	N/A	-	-	-	N/A	-	-	-	0.86	<0.001*
Generalized epilepsy	N/A	-	-	-	N/A	-	-	-	0.61	<0.001*
Anorexia Nervosa	25	-0.07	0.93	0.416	39	0.00	1.00	0.983	0.00	0.953
Anxiety (Case-control)	26	0.08	1.08	0.658	3	0.01	1.01	0.824	0.39	0.036
Anxiety (Continuous)[1]	26	0.00	1.00	0.990	6	-0.05	0.95	0.658	0.05	0.770
Attention-deficit/hyperactivity disorder	25	0.02	1.02	0.766	102	0.02	1.02	0.193	0.23	<0.001*
Autism	25	-0.14	0.87	0.112	21	0.01	1.01	0.747	-0.17	0.059
Bipolar disorder	25	-0.08	0.92	0.423	187	0.00	1.00	0.713	-0.11	0.058
Intelligence	28	-0.01	0.99	0.533	487	-0.13	0.88	<0.001*	-0.24	<0.001*
Depression	25	0.05	1.05	0.169	174	0.02	1.02	0.447	0.10	0.102
Obsessive-compulsive disorder	25	0.21	1.23	0.339	7	-0.03	0.97	0.078	-0.06	0.609
Schizophrenia	25	-0.15	0.86	0.059	489	-0.01	0.99	0.274	-0.14	0.005
Suicide attempts	24	-0.03	0.97	0.730	29	0.00	1.00	0.952	0.12	0.209
Tic-disorders (Tourette Syndrome)	25	0.24	1.27	0.128	9	0.00	1.00	0.814	-0.0103	0.925

[1]In our primary analysis, we leverage the case-control anxiety definitions, to ensure that the findings reflect manifested anxiety. However, we chose to also analyse anxiety as a continuous score obtained from the original GWAS to enhance statistical efficiency.

*Statistically significant at Bonferroni correction (α=0.00075758 for MR and α=0.001515 for LDSC).

Supplemental Table 5. Extended results from the LDSC and MR analyses of generalized epilepsy and selected psychiatric phenotypes.

Generalized epilepsy										
	Effect of epilepsy on comorbidity				Effect of Comorbidity on Epilepsy				Genetic correlation	
**Phenotype**	**N_{iv}**	**β**	**OR**	**P-value**	**N_{iv}**	**β**	**OR**	**P-value**	**rg**	**P-value**
Epilepsy (broadly defined)	N/A	-	-	-	N/A	-	-	-	0.92	<0.001*
Focal epilepsy	N/A	-	-	-	N/A	-	-	-	0.61	<0.001*
Anorexia Nervosa	67	-0.01	0.99	0.839	39	-0.04	0.96	0.251	0.01	0.803
Anxiety (Case-control)	78	0.05	1.05	0.476	3	-0.06	0.94	0.320	0.16	0.137
Anxiety (Continuous)[1]	78	-0.02	0.98	0.151	6	0.02	1.02	0.916	0.05	0.692
Attention-deficit/hyperactivity disorder	67	0.06	1.06	0.071	102	0.06	1.06	0.015	0.10	0.009
Autism	67	0.00	1.00	0.926	21	-0.02	0.98	0.563	-0.09	0.088
Bipolar disorder	67	-0.02	0.98	0.647	187	-0.03	0.98	0.235	-0.10	0.003
Intelligence	80	0.00	1.00	0.906	487	-0.14	0.87	<0.001*	-0.14	<0.001*
Depression	66	0.00	1.00	0.745	174	-0.03	0.97	0.502	-0.01	0.728
Obsessive-compulsive disorder	67	-0.04	0.96	0.658	7	-0.02	0.98	0.442	-0.13	0.061
Schizophrenia	67	0.03	1.03	0.483	489	-0.02	0.98	0.264	-0.07	0.050
Suicide attempts	65	-0.03	0.97	0.365	29	-0.01	0.99	0.779	0.06	0.256
Tic-disorders (Tourette Syndrome)	67	0.02	1.02	0.747	9	-0.01	0.99	0.835	0.02	0.699

[1]In our primary analysis, we leverage the case-control anxiety definitions, to ensure that the findings reflect manifested anxiety. However, we chose to also analyse anxiety as a continuous score obtained from the original GWAS to enhance statistical efficiency.

*Statistically significant at Bonferroni correction (α=0.00075758 for MR and α=0.001515 for LDSC).

eMethods - Mendelian sensitivity analysis

To allow for the potential of horizontal pleiotropy, whereby the instruments are related to the outcome through some pathway that is independent of the exposure, we employed MR-Egger regression.[1] In MR-Egger regression the intercept is unconstrained, in contrast to the IVW regression, and a non-zero intercept can be interpreted as the presence of horizontal pleiotropy and the slope the causal effect in presence of horizontal pleiotropy,[1] under the instrument independent of direct effects (InSIDE) assumption.

In addition to MR-Egger, we conducted a series of commonly employed MR analyses with relaxed instrumental variable assumptions. First, we conducted weighted median MR, which provides an unbiased estimate when at least 50% of the genetic variants are valid instruments. Second, we conducted a mode-based MR which assumes that more instruments estimate the true causal effect of the exposure than any other effect. The benefit of the median and mode-based regressions compared to MR Egger is that they are more efficient – at the cost of being less robust to horizontal pleiotropy.

Since we employed an inclusive approach to instrument selection (p-value<5×10^{-06}), and this may lead to bias towards the null under weak instrument bias and absence of sample overlap between GWAS studies, we repeated our primary analyses (IVW regression) using a more conservative p-value threshold (p-value<5×10^{-08}) for instrument selection.

Findings of mendelian sensitivity analysis

There was no evidence of horizontal pleiotropy (Supplemental Figure 6 & Supplemental Table 6). The MR Egger intercept that had the lowest p-value was the association between genetic liability to bipolar disorder and epilepsy (Egger intercept -0.005, p=0.073). The greatest egger intercept was identified for the association between anxiety disorder and generalized epilepsy (Egger intercept 0.089), although its standard error was large (p=0.738).

There was no consistent difference, at large, between our primary causal analyses and several sensitivity analyses (Supplement Supplemental Table 7). Specifically, using the weighted median, egger, simple mode, and weighted mode regression was consistent with the IVW regression (primary analyses). As in our primary analyses, there was a persistent negative effect of genetic liability to intelligence on epilepsy when employing median and mode-based analyses (Supplement Supplemental Table 7).

There was no difference between our primary analysis and when considering a more conservative p-value threshold (p-value<5×10^{-08}) for instrument selection, although the precision was reduced.

However, one deviation from our primary analysis is noteworthy. In our initial assessment, we identified a positive association between genetic predisposition to (broadly classified) epilepsy and anxiety. However, this association could not be reproduced when employing the continuous anxiety score or utilizing MR-Egger.

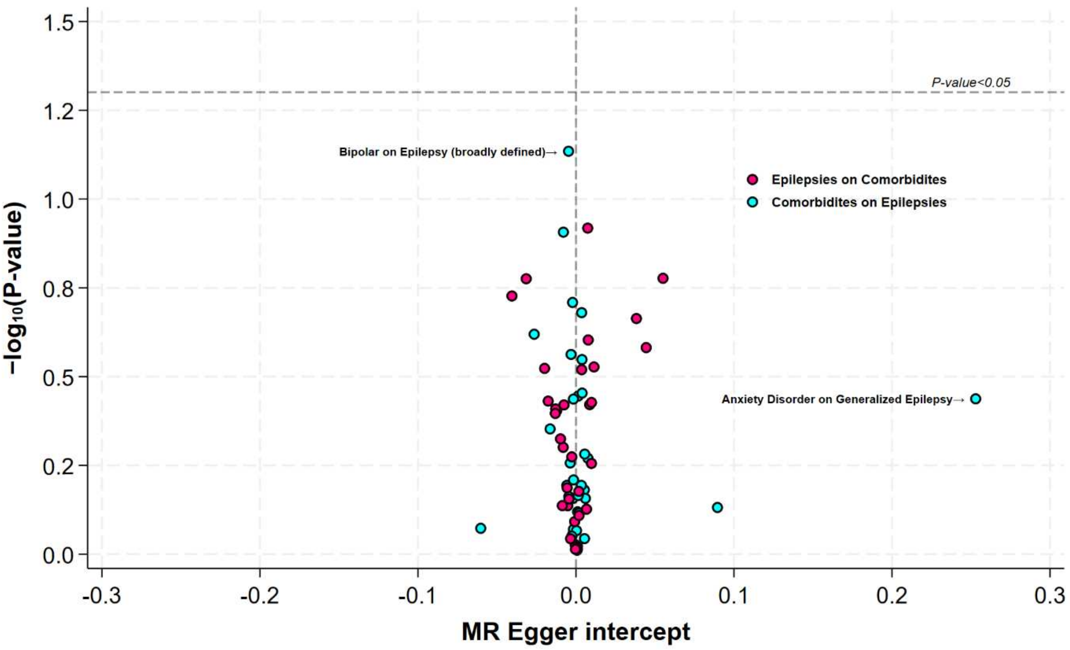

Supplemental Figure 6. Volcano plot of the MR-Egger intercept and corresponding intercept p-value for each Mendelian randomization analysis. Departure from zero of the egger-intercept is indicative of horizontal pleiotropy, which biases standard Mendelian randomization analysis. The grey dotted line horizontally marks the zero intercept and vertically marks the level above which a p-value is less than 0.05 on the standard scale. The mendelian analysis of genetic liability to bipolar on epilepsy is highlighted as the egger intercept with the lowest p-value (0.073269). The mendelian analysis of genetic liability to anxiety disorder on generalized epilepsy is highlighted as it was of the greatest magnitude (although its p-value is large).

Supplemental Table 6. Egger intercepts for all Mendelian analyses.

	Epilepsy on Comorbidity		Comorbidity on epilepsy	
**Epilepsy (broadly defined)**	**Egger Intercept**	**P-value**	**Egger Intercept**	**P-value**
Attention-deficit/hyperactivity disorder	0.011	0.297	0.004	0.283
Anorexia Nervosa	-0.005	0.729	-0.001	0.938
Anxiety (Case-control)	0.038	0.217	0.089	0.738
Anxiety (Continuous)	0.007	0.121	-0.002	0.875
Autism	-0.006	0.650	0.005	0.658
Bipolar disorder	-0.006	0.493	-0.005	0.073
Intelligence	-0.003	0.532	-0.002	0.195
Major depression	0.000	0.953	0.001	0.759
Obsessive-compulsive disorder	-0.041	0.187	-0.006	0.640
Schizophrenia	-0.012	0.394	0.001	0.358
Suicide attempts	-0.008	0.500	-0.002	0.696
Tic-disorders (Tourette Syndrome)	0.007	0.746	0.001	0.974
**Focal epilepsy**				
Attention-deficit/hyperactivity disorder	-0.013	0.390	0.004	0.351
Anorexia Nervosa	0.010	0.555	-0.002	0.850
Anxiety (Case-control)	0.044	0.262	-0.060	0.845
Anxiety (Continuous)	0.008	0.249	0.006	0.696
Autism	0.001	0.964	0.008	0.537
Bipolar disorder	-0.018	0.370	-0.003	0.274
Intelligence	-0.001	0.809	-0.002	0.365
Major depression	0.002	0.778	0.001	0.683
Obsessive-compulsive disorder	0.055	0.167	-0.005	0.687
Schizophrenia	-0.013	0.401	0.000	0.858
Suicide attempts	-0.009	0.729	-0.004	0.553
Tic-disorders (Tourette Syndrome)	-0.003	0.904	-0.003	0.889
**Generalized epilepsy**				
Attention-deficit/hyperactivity disorder	-0.008	0.380	0.003	0.640
Anorexia Nervosa	-0.010	0.473	0.001	0.949
Anxiety (Case-control)	0.024	0.218	0.253	0.365
Anxiety (Continuous)	0.004	0.302	-0.027	0.240
Autism	-0.004	0.698	0.001	0.970
Bipolar disorder	0.009	0.379	-0.008	0.124
Intelligence	0.002	0.665	-0.002	0.617
Major depression	0.000	0.960	0.002	0.765
Obsessive-compulsive disorder	-0.032	0.168	-0.016	0.444
Schizophrenia	0.010	0.374	0.004	0.209
Suicide attempts	0.000	0.968	0.005	0.522
Tic-disorders (Tourette Syndrome)	-0.020	0.300	0.005	0.903

Supplemental Table 7. Mendelian sensitivity analyses (see eMethods for description of each analysis).

	Epilepsy on Comorbidity				Comorbidity on Epilepsy			
	N_{iv}	β	OR	P-value	N_{iv}	β	OR	P-value
Epilepsy (broadly defined)								
Anorexia Nervosa								
MR Egger	37	-0.02	0.98	0.958	39	-0.01	0.99	0.919
Simple mode	37	-0.31	0.73	0.194	39	-0.07	0.94	0.143
Weighted median	37	-0.20	0.82	0.106	39	-0.02	0.98	0.206
Weighted mode	37	-0.18	0.83	0.386	39	-0.07	0.94	0.131
Anxiety (Case-control)								
MR Egger	39	-0.72	0.49	0.449	3	-0.60	0.55	0.733
Simple mode	39	0.98	2.65	0.044	3	-0.05	0.95	0.419
Weighted median	39	0.67	1.95	0.005	3	-0.02	0.98	0.498
Weighted mode	39	0.91	2.48	0.050	3	-0.04	0.96	0.435
Anxiety (Continuous)								
MR Egger	39	-0.18	-	0.225	6	0.08	1.08	0.857
Simple mode	39	0.13	-	0.141	6	0.09	1.09	0.631
Weighted median	39	0.06	-	0.107	6	0.04	1.04	0.727
Weighted mode	39	0.13	-	0.132	6	0.07	1.07	0.690
Attention-deficit/hyperactivity disorder								
MR Egger	37	-0.22	0.80	0.495	102	-0.04	0.96	0.554
Simple mode	37	0.03	1.03	0.872	102	-0.02	0.98	0.743
Weighted median	37	0.04	1.04	0.628	102	0.03	1.03	0.060
Weighted mode	37	0.00	1.00	0.989	102	0.00	1.00	0.937
Autism								
MR Egger	37	0.21	1.23	0.576	21	-0.06	0.95	0.719
Simple mode	37	0.16	1.17	0.539	21	0.05	1.05	0.362
Weighted median	37	0.11	1.12	0.337	21	0.03	1.03	0.231
Weighted mode	37	0.15	1.16	0.526	21	0.04	1.04	0.391
Bipolar disorder								
MR Egger	37	0.05	1.05	0.865	187	0.08	1.08	0.095
Simple mode	37	-0.21	0.81	0.290	187	-0.02	0.98	0.668
Weighted median	37	-0.19	0.83	0.027	187	-0.01	0.99	0.499
Weighted mode	37	-0.28	0.76	0.130	187	-0.01	0.99	0.789
Intelligence								
MR Egger	42	0.05	-	0.698	487	0.01	1.01	0.948
Simple mode	42	-0.02	-	0.807	487	-0.19	0.83	0.070
Weighted median	42	-0.01	-	0.649	487	-0.10	0.91	0.000
Weighted mode	42	0.00	-	0.972	487	-0.11	0.89	0.260
Depression								
MR Egger	36	-0.02	0.98	0.945	174	-0.04	0.96	0.765
Simple mode	36	0.06	1.06	0.491	174	-0.01	0.99	0.951
Weighted median	36	-0.01	0.99	0.874	174	-0.01	0.99	0.795
Weighted mode	36	0.06	1.06	0.421	174	-0.02	0.98	0.869
Obsessive-compulsive disorder								
MR Egger	37	1.06	2.88	0.260	7	0.01	1.01	0.894
Simple mode	37	0.56	1.75	0.400	7	-0.03	0.97	0.347
Weighted median	37	0.29	1.34	0.305	7	-0.03	0.98	0.177
Weighted mode	37	0.55	1.73	0.403	7	-0.03	0.97	0.324
Schizophrenia								
MR Egger	37	0.23	1.26	0.601	489	-0.04	0.97	0.237
Simple mode	37	-0.23	0.80	0.312	489	0.00	1.00	0.939
Weighted median	37	-0.07	0.93	0.404	489	0.00	1.00	0.735
Weighted mode	37	0.15	1.17	0.476	489	0.00	1.00	0.925
Suicide attempts								
MR Egger	36	0.23	1.26	0.529	29	0.04	1.04	0.658
Simple mode	36	-0.05	0.95	0.799	29	0.02	1.02	0.783
Weighted median	36	0.01	1.01	0.948	29	0.01	1.01	0.844
Weighted mode	36	-0.05	0.95	0.797	29	0.02	1.02	0.783
Tic-disorders (Tourette Syndrome)								
MR Egger	37	-0.28	0.75	0.647	9	0.00	1.00	0.993
Simple mode	37	-0.24	0.78	0.600	9	0.02	1.02	0.682
Weighted median	37	-0.19	0.83	0.366	9	0.01	1.01	0.589
Weighted mode	37	-0.22	0.81	0.593	9	0.02	1.02	0.571
Focal epilepsy								
Anorexia Nervosa								
MR Egger	25	-0.32	0.73	0.462	39	0.02	1.02	0.855
Simple mode	25	-0.19	0.83	0.373	39	-0.03	0.97	0.597
Weighted median	25	-0.08	0.92	0.490	39	-0.02	0.98	0.381
Weighted mode	25	-0.17	0.85	0.466	39	-0.02	0.98	0.709
Anxiety (Case-control)								
MR Egger	26	-1.07	0.34	0.301	3	0.41	1.51	0.841
Simple mode	26	-0.59	0.55	0.284	3	-0.03	0.97	0.622
Weighted median	26	-0.16	0.86	0.523	3	-0.01	0.99	0.816
Weighted mode	26	-0.57	0.57	0.334	3	-0.03	0.97	0.628

Anxiety (Continuous)								
MR Egger	26	-0.20	-	0.256	6	-0.25	0.78	0.638
Simple mode	26	-0.02	-	0.802	6	0.16	1.17	0.530
Weighted median	26	0.00	-	0.960	6	-0.04	0.96	0.762
Weighted mode	26	-0.03	-	0.679	6	-0.16	0.85	0.445
Attention-deficit/hyperactivity disorder								
MR Egger	25	0.36	1.43	0.369	102	-0.05	0.95	0.509
Simple mode	25	0.19	1.21	0.327	102	0.07	1.08	0.172
Weighted median	25	0.01	1.01	0.885	102	0.03	1.03	0.094
Weighted mode	25	0.16	1.18	0.380	102	0.06	1.07	0.181
Autism								
MR Egger	25	-0.16	0.85	0.738	21	-0.09	0.91	0.577
Simple mode	25	-0.24	0.79	0.346	21	0.04	1.04	0.546
Weighted median	25	-0.19	0.82	0.123	21	0.01	1.01	0.845
Weighted mode	25	-0.19	0.83	0.470	21	-0.04	0.96	0.488
Bipolar disorder								
MR Egger	25	0.38	1.46	0.468	187	0.06	1.06	0.250
Simple mode	25	-0.20	0.82	0.225	187	-0.01	0.99	0.772
Weighted median	25	-0.14	0.87	0.098	187	-0.01	0.99	0.685
Weighted mode	25	-0.19	0.83	0.234	187	-0.01	0.99	0.745
Intelligence								
MR Egger	28	0.01	-	0.930	487	-0.04	0.97	0.743
Simple mode	28	-0.01	-	0.829	487	-0.14	0.87	0.232
Weighted median	28	-0.01	-	0.683	487	-0.12	0.88	0.000
Weighted mode	28	-0.01	-	0.820	487	-0.14	0.87	0.182
Depression								
MR Egger	25	0.00	1.00	0.991	174	-0.04	0.96	0.790
Simple mode	25	0.07	1.08	0.349	174	-0.05	0.96	0.688
Weighted median	25	0.06	1.07	0.095	174	-0.02	0.98	0.612
Weighted mode	25	0.07	1.08	0.288	174	-0.05	0.96	0.648
Obsessive-compulsive disorder								
MR Egger	25	-1.21	0.30	0.245	7	-0.01	0.99	0.910
Simple mode	25	0.40	1.49	0.569	7	-0.05	0.95	0.186
Weighted median	25	0.16	1.18	0.581	7	-0.03	0.97	0.192
Weighted mode	25	0.37	1.45	0.559	7	-0.01	0.99	0.689
Schizophrenia								
MR Egger	25	0.19	1.21	0.636	489	-0.01	0.99	0.659
Simple mode	25	-0.15	0.86	0.280	489	-0.01	0.99	0.825
Weighted median	25	-0.06	0.94	0.435	489	0.00	1.00	0.874
Weighted mode	25	-0.05	0.95	0.685	489	0.00	1.00	0.934
Suicide attempts								
MR Egger	24	0.20	1.22	0.771	29	0.06	1.06	0.576
Simple mode	24	0.15	1.16	0.447	29	0.06	1.07	0.423
Weighted median	24	0.04	1.04	0.686	29	0.04	1.04	0.286
Weighted mode	24	0.12	1.13	0.513	29	0.06	1.06	0.445
Tic-disorders (Tourette Syndrome)								
MR Egger	25	0.33	1.39	0.662	9	0.02	1.02	0.865
Simple mode	25	0.22	1.24	0.633	9	0.00	1.00	0.970
Weighted median	25	0.21	1.23	0.307	9	0.00	1.00	0.988
Weighted mode	25	0.19	1.21	0.677	9	0.00	1.00	0.969
Generalized epilepsy								
Anorexia Nervosa								
MR Egger	67	0.16	1.17	0.511	39	-0.05	0.95	0.787
Simple mode	67	0.03	1.03	0.813	39	-0.03	0.97	0.721
Weighted median	67	0.05	1.05	0.356	39	-0.02	0.98	0.605
Weighted mode	67	0.05	1.05	0.733	39	-0.03	0.97	0.723
Anxiety (Case-control)								
MR Egger	78	-0.38	0.68	0.279	3	-1.73	0.18	0.355
Simple mode	78	0.03	1.04	0.888	3	0.00	1.00	0.995
Weighted median	78	0.06	1.06	0.540	3	-0.01	0.99	0.875
Weighted mode	78	0.10	1.10	0.640	3	0.00	1.00	0.997
Anxiety (Continuous)								
MR Egger	78	-0.08	-	0.201	6	0.93	2.52	0.243
Simple mode	78	-0.01	-	0.696	6	0.17	1.18	0.517
Weighted median	78	-0.02	-	0.366	6	0.16	1.18	0.402
Weighted mode	78	-0.01	-	0.663	6	0.18	1.20	0.473
Attention-deficit/hyperactivity disorder								
MR Egger	67	0.19	1.21	0.213	102	0.00	1.00	0.987
Simple mode	67	-0.08	0.92	0.495	102	0.05	1.05	0.505
Weighted median	67	0.03	1.04	0.392	102	0.05	1.05	0.066
Weighted mode	67	-0.09	0.91	0.453	102	0.04	1.04	0.569
Autism								
MR Egger	67	0.08	1.08	0.690	21	-0.03	0.97	0.896
Simple mode	67	-0.03	0.98	0.858	21	0.05	1.05	0.596
Weighted median	67	-0.01	0.99	0.905	21	0.04	1.04	0.402
Weighted mode	67	-0.02	0.98	0.905	21	0.04	1.04	0.578

Bipolar disorder								
MR Egger	67	-0.16	0.85	0.338	187	0.11	1.12	0.219
Simple mode	67	-0.07	0.93	0.472	187	-0.04	0.96	0.633
Weighted median	67	-0.03	0.97	0.502	187	-0.04	0.96	0.116
Weighted mode	67	-0.07	0.94	0.481	187	-0.04	0.96	0.646
Intelligence								
MR Egger	80	-0.03	-	0.654	487	-0.05	0.95	0.796
Simple mode	80	-0.03	-	0.408	487	-0.54	0.58	0.005
Weighted median	80	0.00	-	0.750	487	-0.10	0.91	0.033
Weighted mode	80	-0.01	-	0.712	487	-0.55	0.58	0.003
Depression								
MR Egger	66	0.00	1.00	0.986	174	-0.10	0.91	0.679
Simple mode	66	-0.04	0.96	0.347	174	-0.13	0.88	0.429
Weighted median	66	-0.01	0.99	0.551	174	-0.11	0.89	0.027
Weighted mode	66	-0.03	0.97	0.413	174	-0.13	0.88	0.359
Obsessive-compulsive disorder								
MR Egger	67	0.49	1.63	0.213	7	0.05	1.06	0.611
Simple mode	67	-0.14	0.87	0.636	7	-0.07	0.93	0.204
Weighted median	67	-0.12	0.89	0.339	7	-0.04	0.96	0.222
Weighted mode	67	-0.14	0.87	0.582	7	-0.06	0.94	0.215
Schizophrenia								
MR Egger	67	-0.14	0.87	0.471	489	-0.08	0.92	0.136
Simple mode	67	0.08	1.09	0.381	489	-0.09	0.92	0.199
Weighted median	67	0.02	1.02	0.626	489	-0.01	0.99	0.354
Weighted mode	67	0.06	1.06	0.470	489	-0.06	0.94	0.319
Suicide attempts								
MR Egger	65	-0.02	0.98	0.896	29	-0.10	0.91	0.491
Simple mode	65	0.00	1.00	0.955	29	-0.07	0.93	0.464
Weighted median	65	0.01	1.01	0.894	29	-0.05	0.95	0.287
Weighted mode	65	0.00	1.00	0.979	29	-0.07	0.94	0.458
Tic-disorders (Tourette Syndrome)								
MR Egger	67	0.36	1.43	0.279	9	-0.05	0.95	0.884
Simple mode	67	0.07	1.07	0.791	9	-0.09	0.91	0.118
Weighted median	67	0.01	1.01	0.923	9	-0.05	0.95	0.170
Weighted mode	67	-0.13	0.88	0.606	9	-0.09	0.91	0.177

Supplement References

1. Bowden J, Davey Smith G, Burgess S. Mendelian randomization with invalid instruments: effect estimation and bias detection through Egger regression. Int J Epidemiol. 2015;44(2):512-25.
